# N-Back Related ERPs Depend on Stimulus Type, Task Structure, Pre-processing, and Lab Factors

**DOI:** 10.3389/fnhum.2020.549966

**Published:** 2020-10-28

**Authors:** Mahsa Alizadeh Shalchy, Valentina Pergher, Anja Pahor, Marc M. Van Hulle, Aaron R. Seitz

**Affiliations:** ^1^Department of Psychology, University of California, Riverside, Riverside, CA, United States; ^2^Department of Neurosciences, Laboratory for Neuro- and Psychophysiology, KU Leuven, Leuven, Belgium; ^3^Department of Cognitive Neuropsychology, Harvard University, Cambridge, MA, United States

**Keywords:** working memory, N-Back, ERPs, experimental features, cross-laboratory, pre-processing

## Abstract

The N-Back, a common working memory (WM) updating task, is increasingly used in basic and applied psychological research. As such, an increasing number of electroencephalogram (EEG) studies have sought to identify the electrophysiological signatures of N-Back task performance. However, *stimulus type*, *task structure*, pre-processing methods, and differences in the laboratory environment, including the EEG recording setup employed, greatly vary across studies, which in turn may introduce inconsistencies in the obtained results. Here we address this issue by conducting nine different variations of an N-Back task manipulating *stimulus type* and *task structure*. Furthermore, we explored the effect of the pre-processing method used and differences in the laboratory environment. Results reveal significant differences in behavioral and electrophysiological signatures in response to N-Back *stimulus type*, *task structure*, pre-processing method, and laboratory environment. In conclusion, we suggest that experimental factors, analysis pipeline, and laboratory differences, which are often ignored in the literature, need to be accounted for when interpreting findings and making comparisons across studies.

## Introduction

Working memory (WM), defined as a limited capacity system responsible for temporary storage and manipulation of relevant information (Baddeley, 2012), has been studied extensively in the last few decades because it correlates with a wide range of complex cognitive abilities such as problem-solving, reasoning, learning and planning of goal-directed behaviors (Miyake and Shah, 1999). A considerable number of studies have addressed behavioral and neurophysiological, and underlying hypothetical constructs of WM using both single session (Scharinger et al., 2015, 2017) and repeated practice (Anguera et al., 2012; Buschkuehl et al., 2014; Jaeggi et al., 2014).

One of the commonly used techniques to probe WM is the N-Back task, a complex task that requires storage, maintenance, and manipulation of information (Chen et al., 2008; Jaeggi et al., 2008) as well as inhibitory and interference control (Oberauer, 2005; Kane et al., 2007). The N-Back task has been used in single-session behavioral (Jaeggi et al., 2010; Brouwer et al., 2012) and neurophysiological (Krause et al., 2000; Pesonen et al., 2007; Esposito et al., 2009; Scharinger et al., 2017) studies as well as in multi-session behavioral (Jaeggi et al., 2008, 2014; Minear et al., 2016; Blacker et al., 2017) and neurophysiological (Chen and Mitra, 2009; Dong et al., 2015; Pergher et al., 2018) training studies, to name a few. Many N-Back studies focus on task difficulty at different N-levels, indicating lower ERP amplitudes for more difficult tasks (Brouwer et al., 2012; Herff et al., 2014; Scharinger et al., 2017; Pergher et al., 2019b) and/or *stimulus type*, such as the use of spatial (for instance when the target stimulus occurs in different locations on the screen) vs. verbal (for instance when the presented stimulus is word or syllable) stimuli. This indicates that stimulus and load factors play a significant role in modulating P2, N2, and P3 components (Ross and Segalowitz, 2000; Polich, 2007; Chen et al., 2008; Chen and Mitra, 2009). However, there are many other task parameters such as stimulus duration, inter-stimulus interval (ISI), feedback, etc. that, although previously explored, are rarely consistent across N-Back studies (for a review see Pergher et al., 2019a). Different combinations of these parameters may differentially affect electrophysiological signatures associated with task performance and thus limit the interpretation of the functional significance of ERP components related to the N-Back task and their comparison across studies.

Here we examine several candidate factors that may affect ERP and behavioral signatures during N-Back task performance, not only in terms of task parameters such as *stimulus type* (*words*, *pictures, and colors*) and (stimulus duration, ISI, and feedback) but also in terms of different data pre-processing pipelines and laboratory effects, such as differences in room setup, computer testing stations, as well as electroencephalogram (EEG) hardware and software. While this is true of numerous areas of ERP research, the N-Back is particularly notorious in how it varies across studies (Owen et al., 2005; Kane et al., 2007; Mencarelli et al., 2019) and the data presented here is the first to detail the extent of these efforts for a variety of N-Back variations.

## Materials and Methods

Three datasets involving the N-Back task were included in the current study. Dataset I was collected specifically for the current study and was collected at the University of California—Riverside (UCR), USA. The purpose of this study was to explore the potential factors that affect ERP morphology and behavioral signatures of the N-Back task and to replicate experimental procedures described in two published datasets collected in different labs (Datasets II and III). Dataset II was collected at KU Leuven, Belgium (Pergher et al., 2018) as part of a study that investigated near and far transfer effects, the former involving cognitive sub-processes similar to the one practiced during training, whereas the latter calling upon other mental processes (De Ribaupierre and Lecerf, 2006), after 10 N-Back training sessions using behavioral and EEG recording. Dataset III was collected at the University of Maribor (UM), Slovenia (Pahor and Jaušovec, 2018) in a study that examined the effects of transcranial alternating current stimulation (tACS) on WM performance and EEG responses. Participants in each dataset were healthy young subjects, who reported normal or corrected-to-normal vision, no history of psychiatric or neurological diseases, and were not taking any medication known to interfere with cognitive functioning.

### Dataset I: UCR

#### Participants

Thirty-six right-handed adults (27 females and nine males, mean age = 19.58, SD = 0.97), undergraduate students, were recruited from UCR. The experimental protocol was approved by the Institutional Review Board of UCR and all participants gave their informed consent before the experiment. They received course credit and a payment of $10 for participating in two sessions.

#### Stimuli and Task Structure

**Figure 1:**
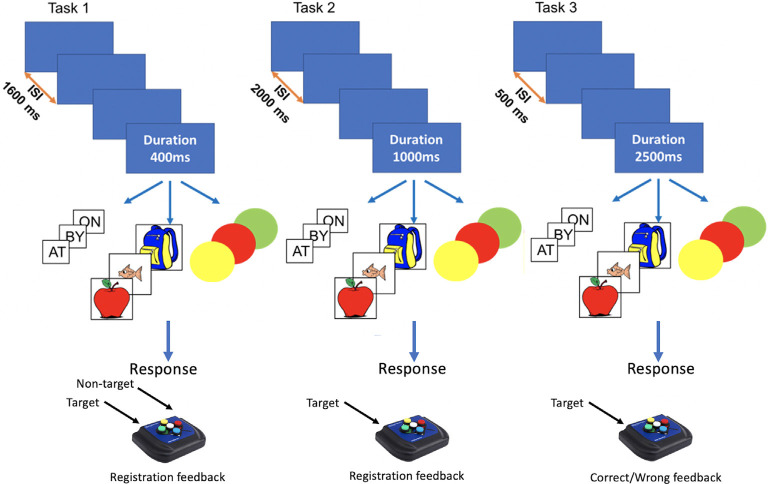
Graphic rendition of N-Back task features for *stimulus type*, stimulus duration, and Inter-stimulus Interval (ISI) for Dataset I.

Nine variants of the N-Back task were obtained by crossing three *task structures* (see below) with three *stimulus types*: *words* (i.e., so, do, up), *pictures* (i.e., apple, fish, and bag), and *colors* (i.e., red, green, and blue). *Task structures* differed in terms of stimulus duration, ISI, response contingency, and feedback (see Figure 1) and were modeled after tasks used in previous studies, as mentioned above: *task 1* (Pahor and Jaušovec, 2018), *task 2* (Pergher et al., 2018), and *task 3* (Mohammed et al., 2017).

*Task 1* had a stimulus duration of 400 ms, ISI of 1,600 ms, and employed a two-alternative forced-choice design for responding to targets and non-targets during the ISI. A white fixation cross appeared during ISI, turning blue when a response was registered or red if no response was detected. *Task 2* had a stimulus duration of 1,000 ms and ISI of 2,000 ms. During the ISI, participants viewed a white fixation cross and were instructed to press a button only for target trials. *Task 3* had a stimulus duration of 2,500 ms and ISI of 500 ms. Participants were instructed to respond to targets during stimulus presentation and were given feedback for correct (green circle around the stimulus) and incorrect responses (red circle around the stimulus). For *task 1* and *task 2* no response was allowed during stimulus presentation.

#### Procedure

Each participant performed four out of the nine N-Back variations across two different sessions conducted on different days, where the same difficulty levels were administered each day, for a total of approximately 90 min per session. This ensured that all combinations of conditions existed in a within-subject design, even though not all participants completed every condition. The assignment of each participant to each N-Back variant was done randomly based on the subject number to ensure an equal number of participants (*N* = 16) in each variant. Each session consisted of 11 blocks presented in the following order: 1-back practice block, 2-back practice block, four 2-back test blocks, 3-back practice block, and four 3-back experimental blocks. Instructions were provided before each new N-level and 15-s breaks were given between blocks. Practice blocks consisted of sixteen trials during which the participant performed *task 3* with Color stimuli whereas experimental blocks consisted of N + 40 trials (i.e., 2-Back had 42 trials).

The experiment took place in an electrically shielded room with DC lighting. An Apple Mac Mini with OS X 10.6.8 running MATLAB 2007b (Mathworks, Natick, MA, USA) and Psychtoolbox Version 3.0.8 were used to present the task and generate the stimuli (Brainard, 1997; Kleiner et al., 2007). The stimuli were displayed on a 22.5-inch wide Sony Trinitron (Sony Corp., Tokyo, Japan) CRT monitor with a resolution of 1,280 × 1,024 pixels and a refresh rate of 75 Hz. Also, to guarantee temporal precision of event-markers with experimental stimuli, a DATAPixx stimulus unit was used (VPixx, Vision Science Solutions, Saint-Bruno, QC, Canada) that ensured that triggers were sent precisely at the times of the vertical interrupt of the monitor and button presses.

#### EEG Recording

EEG was recorded continuously using a Biosemi Active Two system (Biosemi B.V. Amsterdam) operating at a sampling rate of 2,048 Hz. Active Two system stored the EEG signal with no high-pass filter and low-pass filtered only by the anti-aliasing filter. Thirty-two active Ag/AgCl electrodes placed according to the 10/20 system (Jasper, 1958) at O1, Oz, O2, PO3, PO4, P7, P3, Pz, P4, P8, CP5, CP1, CP2, CP6, T7, C3, Cz, C4, T8, FC5, FC1, FC2, FC6, F7, F3, Fz, F4, F8, AF3, AF4, Fp1, Fp2. Also, six external electrodes were placed on the mastoids for referencing, and to record the horizontal and vertical electrooculogram (EOG).

### Dataset II: KU Leuven

#### Participants

Twenty-three healthy adults (12 females and 11 males, mean age = 24.37, SD = 1.78) were recruited *via* advertisements and flyers^1^. We randomly selected 16 subjects out of the first two sessions of dataset II to have a comparable sample size for cross-laboratory comparison purposes (see Table 1). Before starting the experiment, all participants were informed about the experimental procedure and signed informed consent. They received a payment of 20 euros for participating in two experiments. The study was approved by the UZ KU Leuven ethical committee (S59475).

**Table 1:** Demographics. Means [±Standard Deviations (SDs)] age of participants.

	Participants		
	UCR (Dataset I)	KU Leuven (Dataset II)	UM (Dataset III)
N	36	16	16
Age	19.58 ± 0.97	23.42 ± 0.98	20.56 ± 1.59
Sex	27 F (8 M)	9 F (7 M)	16 F

#### Stimuli and Procedure

Dataset II had a *task structure* similar to *task 2* of Dataset I, mentioned above, where each stimulus was presented for 1,000 ms, followed by an ISI of 2,000 ms. The stimuli were generated using MATLAB 2007b (Mathworks, Natick, MA, USA) and Psychtoolbox Version 3.0.8 (Brainard, 1997; Kleiner et al., 2007) and displayed on a CRT monitor. Participants had to respond only to targets. The stimuli used were pictures (Pergher et al., 2018).

#### EEG Recording

EEG was recorded at full bandwidth with a SynAmpsRT device (Compumedics, Australia) at a sampling rate of 2,000 Hz, using 32 Ag/AgCl electrodes placed at O1, Oz, O2, PO3, P8, P4, Pz, P3, P7, TP9, CP5, CP1, CP2, CP6, TP10, T7, C3, Cz, C4, T8, FC6, FC2, FC1, FC5, F3, Fz, F4, AF3, AF4, Fp1, Fp2. The reference was placed at AFz and the ground at CPz. Also, four external electrodes around the eyes were used for EOG recording following the instructions of Croft and Barry (2000).

### Dataset III: UM

#### Participants

Seventy-two healthy adults were recruited from the University of Maribor, Slovenia (Pahor and Jaušovec, 2018)^2^, 24 of which were assigned to sham stimulation in the first session (all females, mean age = 20.42, SD = 1.56) and were thus not exposed to any active stimulation. Sixteen of these participants (session 1 data only) were randomly selected for Dataset III (see Table 1). The protocol was approved by the Commission for Ethics in Research at the Faculty of Arts. Participants gave written informed consent and received course credit as compensation.

#### Stimuli and Procedure

Dataset III had a *task structure* similar to *task 1* of Dataset I, where each stimulus was shown for 400 ms, followed by an ISI of 1,600 ms. The stimuli were generated on STIM2 (Compumedics Neuroscan Systems, Charlotte, NC, USA) and displayed on a CRT monitor. Participants had to respond to both targets and non-targets. Two types of stimuli were used: two-letter words and colors (Pahor and Jaušovec, 2018).

#### EEG Recording

EEG was recorded over 19 scalp locations based on the 10-20 Electrode Placement System using a Quik-Cap (Quik-Cap Compumedics Neuromedical supplies, Charlotte, NC, USA) with sintered electrodes. The EEG data were recorded using a SynAmps RT system and had a band-pass filter of 0.15–100.0 Hz. The 19 EEG traces were sampled online at 1,000 Hz. Vertical eye movements were recorded using two external electrodes placed above and below the left eye and a ground electrode was applied to the forehead. Two ear lobe references (A1 and A2) were used for online referencing, followed by common average re-referencing.

### Preprocessing and Analysis

#### ERP Pre-processing Pipelines

Two pre-processing pipelines were used to analyze Dataset I: pipeline I and pipeline II. For ERP comparison across the three datasets, only pipeline I was used. The pipelines were chosen as they represented different, but standard approaches to ERP analysis (Croft and Barry, 2000; Delorme and Makeig, 2004; Groppe et al., 2009).

##### Pipeline I

Pipeline I was conducted in EEGLAB (MATLAB 2015a, MathWorks Incorporation; EEGLAB v. 14.1.1 Delorme and Makeig, 2004): the data were resampled to 512 Hz and filtered using a Butterworth filter with lower and upper cut-off frequencies of 0.1 Hz and 40 Hz. Electrode recordings were re-referenced to the average of the mastoid recordings (average mastoid reference, TP9 and TP10). Manual inspection was first performed to locate and remove visible disturbances in the data. Epochs were created from 1,000 ms before to 2,000 ms after stimulus onset, and the pre-onset average was subtracted from the post-onset signal (baseline correction). Independent components analysis (ICA) was used to extract blinking and eye movements within the data. Independent components (ICs) that were identified by the data analyst as ocular artifacts were rejected. Finally, epochs were averaged for each N-Back variant and baseline corrected using 200 ms before stimulus onset.

##### Pipeline II

Pipeline II was conducted by using MATLAB R2016a (Mathworks, Natick, MA, USA). The data were resampled to 1,000 Hz and filtered in the 0.1–30 Hz range using a zero-phase 4th-order Butterworth filter. All electrodes were re-referenced offline to the average of the two mastoid signals (average mastoid reference, TP9 and TP10; Luck, 2014). Epochs were created from 200 ms before to 1,000 ms after stimulus onset, and baseline correction was performed by subtracting the average of the 200 ms pre-stimulus onset signal from the 1,000 ms post-stimulus onset signal. The EOG recorded before and during the experiment was used for correcting the EEG signal for eye artifacts using Croft and Barry’s aligned-artifact average (AAA) procedure (Croft and Barry, 2000). Finally, epochs with EEG signals greater than 50 μV were also excluded as they could signify motion artifacts (van Vliet et al., 2015; Wittevrongel and Van Hulle, 2016). This Pipeline has been developed by the computational neuroscience group at KU Leuven (van Vliet et al., 2014, 2015; Wittevrongel and Van Hulle, 2016) and since then used in dozens of published studies from this group (http://lirias.kuleuven.be/cv?Username=U0013308). The method was developed as it accounts for eye artifacts using an automatic procedure (AAA procedure in Croft and Barry, 2000) rather than having to rely on a *post hoc* ICA analysis where the data analyst needs to identify which IC’s contain those artifacts (as in EEGLAB).

### Statistical Analysis

To assess the effect of N-Back task variations on behavioral responses (average of correct responses across trials) and ERP morphology (we considered the same three midline location electrodes: Fz, Cz, and Pz investigated by Watter et al. (2001), we used nonparametric permutation-based tests (Guo and Yuan, 2017; Derrick et al., 2019) as our datasets failed the Shapiro–Wilk test of normality (Shapiro and Francia, 1972) and the Levene test of equality of variances (Levene, 1960). Specifically, Dataset I utilized a mixed within/between design where each participant performed four out of nine variations. The rationale for using a mixed design was to obtain enough power—16 participants—for each of the nine variations by recruiting only 36 subjects. Therefore, we used a nonparametric permutation-based test to account for the mixed (within/between) design (Efron and RJ, 1993; Farrell et al., 1998). The null hypothesis distribution is generated empirically regardless of any assumptions about the data distribution. The observed results were then assessed relative to the empirical null hypothesis distribution (Collingridge, 2013) and the *p*-value was calculated by comparing the absolute distance between observed values of two groups to the absolute of the empirical null distribution (Cohen, 2017). The results were considered statistically significant when the *p*-value was less than 0.05. We ran 30.000 iterations for permutation testing of behavioral data and 3.000 for ERP data. We adopted the same statistical tests for the comparison between datasets (UCR, KU Leuven, and UM), and ERP and behavioral data comparisons respectively. We note that this *p*-value is monotonically relatable to other measures of reliability, such as differences in signal to noise ratio (SNR).

**Figure 2:**
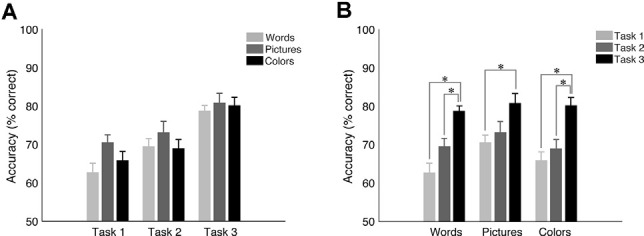
Mean accuracy and SEM for target trials in the University of California—Riverside (UCR) dataset. **(A)** Accuracy as a function of task type. **(B)** Accuracy as a function of *stimulus type*. *Indicates the significance of *p* < 0.05.

Furthermore, we performed a power analysis for accuracy to ensure that our samples, considering the significant results of Figure 2, were large enough. Here, we reported the comparison between *task 1* and *task 3* for *words* that revealed that 14 subjects were sufficient to support the power of 80%, for *colors* that showed that 16 subjects were sufficient to support the power of 80%, and for *pictures* that demonstrated that 22 subjects were sufficient to support the power of 80%. Although the latter did show that a bigger sample size would be necessary, we believe that it does not significantly affect our results.

#### ERP Components

We investigated the following ERP components in the 0–800 ms post-stimulus time window:

P100 (P1), a positive deflection with a peak around 100 ms after stimulus presentation. It is distributed over the lateral occipital electrodes and reflects the early sensory processing of visual stimuli. P1 latency depends on stimulus contrast, such as luminance or SNR, while its amplitude is modulated by attention (Hillyard et al., 1998) and discrimination processes (Vogel and Luck, 2000).

N100 (N1), a negative deflection that peaks around 100–200 ms after stimulus onset. It has a distribution over the entire scalp, but it peaks earlier over the frontal regions of the scalp. It has been shown that its amplitude is modulated by attention. Larger amplitude is associated with attended stimuli, while smaller is associated with increasing stimulus presentation frequency (Luck et al., 1990). N1 latency is affected by cognitive processing effort: the bigger the effort, the longer the latency (Callaway and Halliday, 1982).

P200 (P2), a positive deflection with a peak of around 150–275 ms after stimulus presentation. It is distributed over the fronto-central and parieto-occipital regions of the scalp, but its maximal is over the frontal area. It is elicited by visual stimuli and modulated by attention (Liu et al., 2013). Its amplitude is suppressed by increasing the attentiveness (Kanske et al., 2011) and more frequent target stimuli (Lu et al., 1992).

N200 (N2), a negative deflection detected around 200–350 ms after stimulus onset. It is distributed over the frontal regions of the scalp and posterior regions in visual attention tasks (Folstein and Van Petten, 2008). N2 component reflects several functions such as stimulus identification, attentional shift, and motor response inhibition (Patel and Azzam, 2005).

P300 (P3), a positive deflection with a peak occurring around 250–600 ms after stimulus onset. It shows a stronger distribution over the centro-parietal electrodes on the scalp. Its amplitude becomes larger with infrequent target stimuli and decreases with habituation and task difficulty. Its latency is modulated by the difficulty in discriminating the target stimulus (Picton, 1992; Polich and Kok, 1995).

N400 (N4), a negative deflection detected between 400–600 ms after stimulus onset. It is typically stronger over centro-parietal regions of the scalp and reflects brain response to semantically meaningful stimuli that can include visual and auditory words, sounds, pictures, and faces (Kutas and Federmeier, 2011). N4 amplitude is affected by priming and frequency of the stimulus (Van Petten and Kutas, 1990).

Positive Late Component (PLC), a positive deflection, with a peak occurring around 500–1,000 ms after stimulus onset. It is most prominent for posterior scalp channels. The PLC amplitude is modulated by stimulus repetition: suppressed for stimuli that have been already presented, and generally larger for new stimuli (i.e., “old-new” effect), in both long- and short-term memory paradigms (Olichney et al., 2000; Danker et al., 2008). PLC is believed to index the top-down allocation of attention in a memory recollection process (Mecklinger, 2000).

## Results

### Effect of Stimulus Type and Task Structure—Dataset I (UCR)

To investigate the effect of experimental features (*stimulus type* and *task structure* type), we performed a nonparametric permutation-based analysis on behavioral and electrophysiological data.

#### Behavioral Results

While *pictures* were associated with the highest accuracy level when holding *task structure* constant (Figure 2A; see Supplementary Tables 1–3 in Supplemental Material for means, standard deviations, and statistics per condition, respectfully), there was no statistically significant effect of *stimulus type* on accuracy (*p* > 0.2 for all conditions other than for *task 1*: *words* vs. *pictures*: *p* = 0.075). On the other hand, results revealed a robust overall effect of *task structure* (see Figure 2B), showing higher accuracy for *task 3* vs. *task 1* (*p* < 0.002 for all *stimulus types*), and for *task 3* vs. *task 2* for *words* (*p* < 0.001) and *colors* (*p* < 0.012) but only a trend for *pictures* (*p* = 0.067). However, there was no statistically significant difference between *task 1* and 2 (*p* > 0.4 for all conditions other for *words* *p* = 0.074). These results show that while there is a highly significant effect of tasks, especially *task 3* vs. the others, that the choice of stimulus has a lesser effect on task performance.

#### ERP Morphology

**Figure 3:**
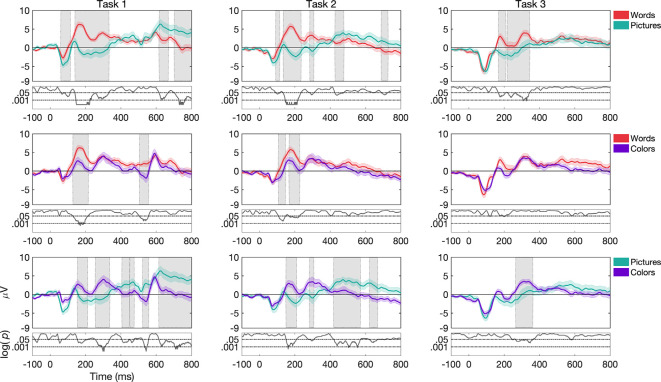
Grand average and SEM of ERP curve for UCR dataset at Cz electrode for target trials during variations of *stimulus types* (*words*, *pictures*, and *colors*). Gray shaded areas indicate significantly different data points (*p* < 0.05). *P*-values that are less than 0.0001 are thresholded to 0.0001 for viewing purposes, as shown by the black curve at the bottom of each graph where log *p*-values are reported.

**Figure 4:**
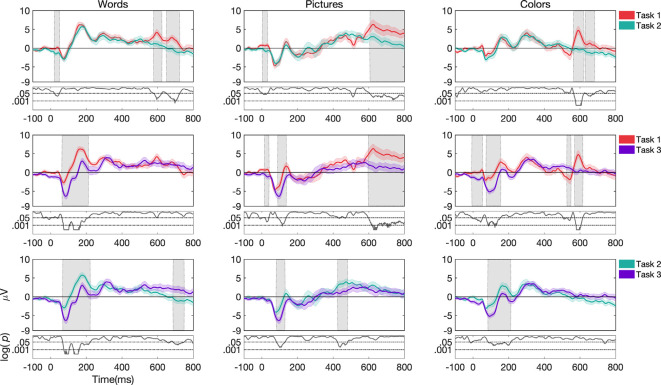
Grand average and SEM of ERP curve for UCR dataset at Cz electrode for target trials during variations of *task structure* (*task 1*, *task 2*, and *task 3*). Gray shaded areas indicate significantly different data points (*p* < 0.05). *P*-values that are less than 0.0001 are thresholded to 0.0001 for viewing purposes.

Overall, ERP morphologies changed substantially both as a function of *stimulus type* and *task structure*. This can be seen in Figures 3, 4 for channel Cz, while channels Fz and Pz are shown in Supplemental Material (Supplementary Figures 1–4). We also presented topographies and reported differences between them in the Supplemental Material (see Supplementary Figure 9 and Supplementary Tables 6, 7). In the following sections, we highlight some of the significant effects by running permutation tests that demonstrate the extent to which differences in morphology across the time-course are different as a function of condition. Significant differences discussed below are concerning shaded regions in graphs that indicate periods in the ERPs where differences are *p*-value of less than or equal to 0.05 for at least 12 consecutive bins with Δt of 1/512 Hz.

##### Effects as a Function of *Stimulus Type*

For *task 1*, ERP morphologies differed more frequently for *pictures* compared to *colors* and *words*, as seen in Figure 3. While *pictures* vs. *words* differed more frequently in the N1, N2 and P2 components in channels Fz, Cz, and Pz (the latter only for P2), *pictures* vs. *colors* showed differences in the N2, P2, and P3 components in channels Fz and Cz. Additionally, *words* vs. *colors* showed differences in the P2 component in channels Fz, Cz, and Pz.

For *task 2*, we found that ERP morphologies differed more frequently for *colors* compared to *pictures* and *words* (see Figure 3). While both *colors* and *words* differed from *pictures* more frequently in the N1 component in channels Cz, Pz, and Fz respectively, *colors* vs. *words* and *colors* vs. *pictures* showed differences mostly in the P2 component in channels Fz and Cz. Additionally, *words* differed from *pictures* and *colors* in the N2 component for channel Cz, while *colors* compared to *pictures* differed more frequently in the P3 component for channels Fz and Cz.

For *task 3*, ERP morphologies differed more frequently for *words* compared to *pictures* (see Figure 3). *words* and *pictures* showed differences in the N2, P2, and P3 components in channel Fz and Cz. Additionally, *words* differed from *colors* in the P3 component in channel Fz and Cz.

##### Effects as a Function of Task Structure

For *words*, ERP morphologies differed more frequently for *task 3* compared to *task 1* and *task 2* (see Figure 4). While *task 3* and *task 1* showed differences in the N1, P1, and P2 components in channels Fz, Cz, and Pz, *task 3* and *task 2* showed differences in the N1 and P2 components in channels Fz, Cz, and Pz. We do note, that in the case of where the stimulus offset occurred at 400 ms, waveforms after 400 ms may have been impacted by a stimulus offset event in addition to other task-related factors.

For *pictures*, ERP morphologies differed more frequently for *task 1* compared to *task 3* and *task 2* (Figure 4). While *task 1* and *task 3* showed differences in the N1 component in channels Fz and Cz, *task 1* and *task 2* showed differences in the P2 component in channels Fz, Cz, and Pz. Additionally, *task 3* differed from *task 2* in the N1 component in channels Fz and Cz.

For *colors*, ERP morphologies differed more frequently for *task 3* compared to *task 1* and *task 2* (Figure 4). While *task 3* and *task 1* showed differences in the N1 and P2 components in channels Fz, Cz, and Pz, *task 3* and *task 2* showed differences in the N1 and P2 components in channels Cz and Pz.

##### Effects as a Function of Task Load and Performance

**Figure 5:**
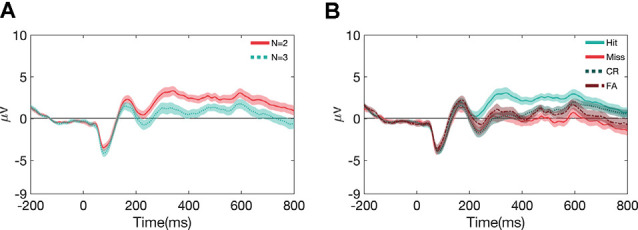
Grand average and SEM of ERP curve for UCR dataset at Cz electrode as a function of N-back load **(A)** and performance metrics **(B)**.

To understand how other factors may have influenced the ERPs, we also examined the effects of memory load and performance on ERP waveforms (see Figure 5). Concerning N-back load (*N* = 2, *N* = 3), the main effect of the load is shown in Figure 5A with this effect of load being significant (*p* < 0.05) for all the components mentioned in this paper except for P1 (see Supplementary Table 4 for stats). However, this effect was largely independent of the *task*, and *stimulus* types (see Supplementary Figure 5, Supplementary Table 4 for the break-down of ERPs and stats across the different *task and stimulus* conditions). Likewise, we also observed differences in the ERPs as a function of metrics of performance (Figure 5B); hits (correctly responded targets), misses (incorrectly responded targets), correct rejections (correctly responded non-targets), and false alarms (incorrectly responded non-targets). There is a significant main effect of performance (*p* < 0.001) for all the components, except for P1. However, again, this effect was largely independent of the *task*, and *stimulus* types (see Supplementary Figure 6, Supplementary Table 5 for ERPs for break-down of ERPs and stats across the different *stimulus* and *task* conditions).

### Comparison Between Pre-processing Pipelines in Dataset I (UCR)

**Figure 6:**
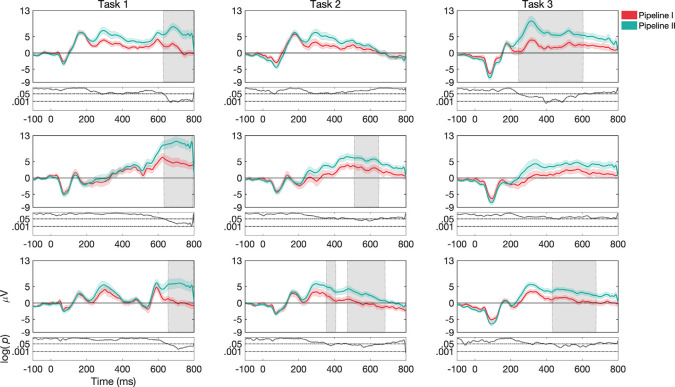
Grand average and SEM of ERP curve at Cz electrode for target trials for different pipelines (Pipeline I vs. Pipeline II) for the UCR dataset (see Supplementary Material for Fz and Pz, Supplementary Figures 7, 8). Gray shaded areas indicate significantly different data points (*p* < 0.05). *P*-values that are less than 0.0001 are thresholded to 0.0001 for viewing purposes. Data in Pipeline II was up-sampled to 512 to make the comparison possible.

We next examined the extent to which differences in analysis pipelines used across labs resulted in changes in estimated ERP morphologies. Interestingly, early ERP components are relatively preserved across the pipelines, but that later ERP components showed significant differences between pipeline I and pipeline II (see Figure 6). Further, these differences showed some interaction with task and stimulus. For example, the effect of the pipeline was found in all variations in *task structure* 1 (for channels Fz, Cz, and Pz). Moreover, the *word* N-Back variation with *task structure* 1 showed significant differences in P3 components between the two pipelines. For *task structure* 2 and *words*, Cz showed a significant difference in N2 and P3 components. For *task structure* 2 and *pictures*, Fz revealed significant differences in PLC and Cz in P3 and PLC. For *task structure* 2 and *color* stimulus, Fz showed significant differences in P3, N4, and PLC signatures and Cz in P3 and PLC. For t*ask structure* 3 and *words*, Fz showed significant differences in N2, P3, N4, and PLC components. Further, Cz showed differences for N2 and P3 and Pz for PLC. For *task structure* 3 and *pictures*, Fz and Cz showed a significant difference in PLC. Finally, for *task structure* 3 and *colors*, Fz showed significant differences in P3, N4, and PLC components and Cz showed a significant difference in PLC.

Because Pipelines I and II differ in several ways ranging from analysis toolbox, eye artifacts removal to reference electrodes, etc., there are too many candidate parameters to be causally related to a specific difference in an ERP component. Nevertheless, these results are interesting as they highlight how the use of different pre-processing pipelines commonly used in the EEG literature can affect ERP morphology at an aggregate level, and in particular, the choice of the pipeline can impact the extent to which one correctly/incorrectly determines differences between conditions. While it would be interesting to unveil possible causal relations between these differences in the pipeline, the goal of the present study is to illuminate the impacts of common methodological differences between studies rather than to fully explain such differences, which would require a larger study. Furthermore, considering the few existing studies in the literature (Dong et al., 2019; Jiang et al., 2019; Yao et al., 2019) that demonstrated a significant role played by pre-processing factors, we think it is likely that the eye artifacts removal method and reference electrodes might have greatest impacts in our pipelines on the resulting ERPs. Still, we note that our analysis of pipeline is merely illustrative of how the pipelines used in the previously published versions of these datasets give rise to different ERP morphologies and that a complete characterization of how pipeline elements affect the signal and/or SNR (Robbins et al., 2020) is beyond the scope of the present manuscript.

### Laboratory Effects

Another potential aspect of variation is the experimental location resulting in behavioral and ERP morphology differences. Specifically, we refer to different laboratories to explore differences in several characteristics such as lab settings, stimuli, tasks, subject pools, subject instructions, processing pipelines, and so on. Using pipeline I, we compared *task 2* (*pictures* only, *N* = 16) as used in Dataset I (UCR) and Dataset II (KU Leuven), as well as *task 1* (*words* only, *N* = 16) which was used in Dataset I (UCR) and Dataset III (UM). We did not compare Datasets II and III as the stimuli were different: *pictures* vs. *words* respectively, whereas Dataset I included both *words* and *pictures* and could therefore be compared to both datasets. We note, that while this analysis is far from comprehensive and it would be ideal to collect data on identical procedures across the labs, however, this is at least illuminative of other, unexplained, variance that can be expected from different labs conducting similar research but not coordinating on the exact details of the studies, which is typical of the extant literature.

#### Dataset I vs. Dataset II (UCR vs. KU Leuven)

**Figure 7:**
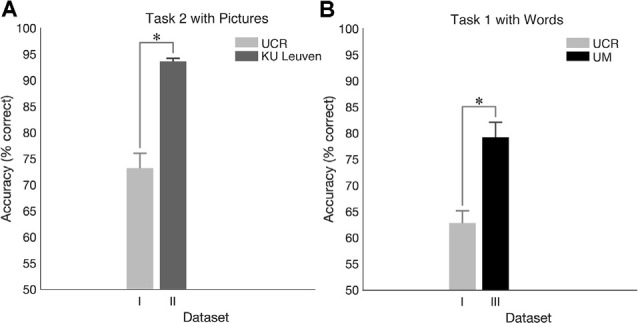
Cross-laboratory accuracy comparison. **(A)** Accuracy for N-Back *task 2* with *pictures* in the dataset I (UCR) and in dataset II (Ku Leuven). **(B)** Accuracy for N-Back *task 1* with *words* in the dataset I (UCR) and in dataset III (University of Maribor, UM). *Indicates significance of *p* < 0.05.

**Figure 8:**
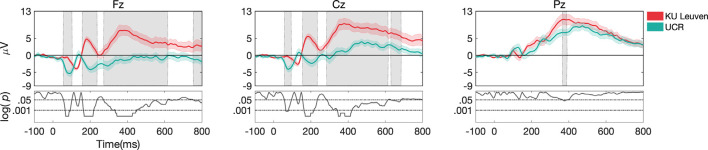
ERP responses during *task 2*, only for target stimuli recorded at different laboratories. Gray shaded areas show significant differences at *p* < 0.05. Both datasets were pre-processed with pipeline I.

Behavioral results for *task 2* showed significantly higher accuracy in Dataset II compared to Dataset I (*p* < 0.001; Figure 7A) and ERP morphology outcomes revealed larger ERP amplitudes in Dataset II compared to Dataset I (Figure 8). Namely, significant differences between Dataset I and Dataset II (*p* < 0.05) were found in P1, N1, P2, N2, and P3 components, in channels Fz and Cz.

#### Dataset I vs. Dataset III (UCR vs. UM)

**Figure 9:**
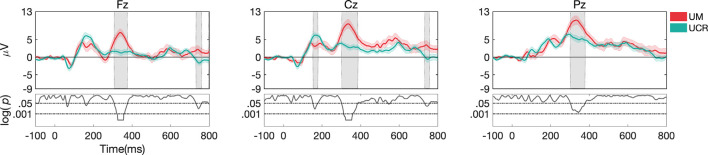
ERP responses during *task 1* (mean and standard deviation of targets) recorded at different laboratories. Gray shaded areas show significant differences at *p* < 0.05. Both datasets were pre-processed with pipeline I.

For *task 1*, higher accuracy was observed in Dataset III compared to Dataset I (*p* < 0.001; Figure 7B), and ERP morphology (Figure 9) indicated significant differences in P1, N1, P2, N2, and P3 components, in channels Fz, Cz, and Pz.

## Discussion

The goal of the present study was to fill a gap in the extant literature by illuminating the extent to which common procedural differences related to N-back task variants, EEG recording setups, and pre-processing pipelines affect behavioral and electrophysiological correlates of performance. To address this, we compared variants of the N-Back task used in three laboratories, two in Europe (Pahor and Jaušovec, 2018; Pergher et al., 2018) and 1 in the US where the behavioral and EEG datasets were replicated. Our findings suggest that *stimulus type*, *task structure*, pre-processing pipeline, and lab factors contribute to differences in behavioral and neurophysiological responses on the N-Back task.

Given the fact that most meta-analyses overlook differences in the N-Back task adopted in each study (Glahn et al., 2005; Heishman et al., 2010; Redick and Lindsey, 2013; Brunoni and Vanderhasselt, 2014), we characterized some of those factors that might affect cognitive task outcomes. First, we examined *task structure* and showed differences in accuracy level between tasks (*task 1*, *task 2*, and *task 3*), revealing higher accuracy for *task 3* compared to the other two, perhaps due to having the longest stimulus duration (2,500 ms) thereby supporting the process for the encoding of information that is facilitated when stimulus duration is longer. Indeed, Kunimi (2016) showed that increasing stimulus duration (from 500 to 5,000 ms) improves memory performance during retention of visuospatial information, whereas Fox et al. (2007) showed that longer ISI was associated with increased accuracy level. We also investigated *stimulus type* and observed better performance for *pictures* of objects compared to *words* and *colors*. In contrast, Nystrom et al. (2000) reported higher accuracy for letters compared to shapes.

Another important aspect when considering the following factors such as *task structure* and *stimulus type* is their impact on ERP morphology. To highlight this variance, we examined differences in several ERP components, named N1, P2, N2, and P3 for both factors, as previous studies suggested ERP component modulation in response to WM experimental features, particularly for *stimulus type*, and observed their spatial distribution. Mecklinger and Pfeifer (1996) reported that the encoding of object features was associated with modulation of P2 component, whereas Ruchkin et al. (1992) showed variations of N2 and P3 components for visuospatial stimuli compared to phonological stimuli, indicating that visuospatial stimuli were processed more quickly than phonological ones. Moreover, Rossion et al. (2003) observed N1 modulation in response to faces and objects compared to objects. Thus while it is clear in the literature that both task and stimulus should influence ERPs in systematic ways, to date this has been largely overlooked in the literature examining ERP signatures of WM tasks such as the N-Back.

In addition to *stimulus type* and *task structure*, we suggest that different experimental laboratories and pre-processing procedures might also affect the accuracy and ERP morphology. Seemingly arbitrary procedures are employed by different laboratories, in terms of environment and equipment, as well as data pre-processed and analyzed by different pipelines, which have been shown to produce different findings (Busch et al., 2006; for review, see Zimmer et al., 2001). Here we show that the same N-Back task performed in two laboratories produces different behavioral and ERP morphology results. However, we suggest interpreting these results carefully, as participants’ individual differences and EEG and analysis operator skills may also have affected these results (Jaeggi et al., 2014). Green et al. (2019) observed that reward, motivation, and/or participant expectations, such as differences in task performance, researcher instructions, etc., could also count as factors for behavioral differences when comparing performances between different laboratories. Moreover, we highlight the impact of pre-processing pipelines on ERP data, supporting the recommendations provided by Smith and Kutas (2015) regarding the power of EEG data pipeline, including baseline correction, artifact rejection, and the filtering procedure (Acunzo et al., 2012) on ERP analysis. In line with the goal of this study, we did not associate a specific step of the pre-processing pipeline procedure to an ERP component or cognitive process since we aimed to show at a more general level the impact of stimuli, tasks, and laboratory environment on both accuracy and ERP responses.

Our study presents several limitations. We considered only accuracy during N-Back performance, because the three Datasets and the related tasks had different response requirements, and so it would have been very complex to compare them. As Dataset I utilized a mixed within/between design, individual differences might have affected ERP signatures attributed to laboratory effects. Indeed, a recent review paper highlighted the variety of features that may impact N-Back performance, including both task and individual features (Pergher et al., 2019a). The samples compared here were of similar age and had a similar educational level (undergraduates) and in Datasets I and II, a similar distribution of gender. While Dataset III only consisted of data collected from females, a recent study by Pliatsikas et al. (2019) demonstrated that gender, age, and education level affect response accuracy after a single N-Back training session in healthy older individuals. Since the present study consists of N-Back performance across 1 or 2 sessions in young subjects, we do expect these variables to have a moderate effect on behavioral and electrophysiological results. Nevertheless, there might be other individual difference factors such as motivation, personality, and WM capacity (Dong et al., 2015) that were not accounted for but could have affected the results. Future studies will need to examine whether these individual differences, along with other factors such as time-of-day and environment, affect N-Back task performance and ERP signatures. Moreover, further studies should also consider the choice of words, pictures, and colors, as they may play an important role in affecting behavioral and ERP responses due to different colors and shapes used, and familiarity with the objects presented. Finally, since Dataset III represents a sham condition in a brain stimulation study (Pahor and Jaušovec, 2018) possibly the placebo effects affected performance. Since we only retained data collected in session 1, i.e., before exposure to active stimulation, it is unlikely that these effects are large. Still, we suggest that while more work can be done to clarify the effects presented here, and that other differences still exist in the extant literature, that the present work is informative of how some of the most common differences in the N-Back between studies can impact observed behavioral and physiological measures.

In conclusion, the present data sets help clarify the extent to which common N-Back task variations in terms of *stimulus type*, *task structure*, and laboratory and processing pipeline give rise to differences in behavioral and physiological outcomes. While future research is needed to help us understand the mechanisms that underly these observed differences, the present work can help readers appreciate effect sizes to be expected related to the many variations considered here. We note that while, in general, it is well acknowledged any difference between studies can have an impact, the significance of these variations in the case of the N-Back has been largely overlooked, thus limiting understanding of their role in affecting accuracy and ERP morphology and of potentially important information related to the mechanisms that regulate WM processes. We suggest that for the field to move forward, experimental features, analysis pipeline, and laboratory differences need to be taken into consideration when interpreting findings and making comparisons across studies.

## Data Availability Statement

The raw data supporting the conclusions of this article will be made available by the authors, without undue reservation.

## Ethics Statement

The studies involving human participants were reviewed and approved by Institutional Review Board of the University of California Riverside. The patients/participants provided their written informed consent to participate in this study.

## Author Contributions

VP, MS, and AS conceived the presented idea and developed the theory. MS, VP, and AP carried out the experiment. MS and VP performed the computations. All authors contributed to the article and approved the submitted version.

## Conflict of Interest

The authors declare that the research was conducted in the absence of any commercial or financial relationships that could be construed as a potential conflict of interest.

## Footnotes

^1^Eight of these participants were included in N-Back training study conducted by Pergher et al. (2018).

^2^Dataset 3 only included participants that were in a sham stimulation condition during their first session.

## References

[B1] Acunzo D. J. MacKenzie G. van Rossum M. C. (2012). Systematic biases in early ERP and ERF components as a result of high-pass filtering. J. Neurosci. Methods 209, 212–218. 10.1016/j.jneumeth.2012.06.011 22743800

[B2] Anguera J. A. Bernard J. A. Jaeggi S. M. Buschkuehl M. Benson B. L. Jennett S.. (2012). The effects of working memory resource depletion and training on sensorimotor adaptation. Behav. Brain Res. 228, 107–115. 10.1016/j.bbr.2011.11.040 22155489 PMC3264800

[B3] Baddeley A. (2012). Working memory: theories, models, and controversies. Annu. Rev. Psychol. 63, 1–29. 10.1146/annurev-psych-120710-100422 21961947

[B4] Blacker K. J. Negoita S. Ewen J. B. Courtney S. M. (2017). N-back versus complex span working memory training. J. Cogn. Enhanc. 1, 434–454. 10.1007/s41465-017-0044-1 29430567 PMC5805159

[B5] Brainard D. H. (1997). The psychophysics toolbox. Spat. Vis. 10, 433–436. 10.1163/156856897x00357 9176952

[B6] Brouwer A. M. Hogervorst M. A. Van Erp J. B. Heffelaar T. Zimmerman P. H. Oostenveld R. (2012). Estimating workload using EEG spectral power and ERPs in the N-back task. J. Neural Eng. 9:045008. 10.1088/1741-2560/9/4/045008 22832068

[B7] Brunoni A. R. Vanderhasselt M. A. (2014). Working memory improvement with non-invasive brain stimulation of the dorsolateral prefrontal cortex: a systematic review and meta-analysis. Brain Cogn. 86, 1–9. 10.1016/j.bandc.2014.01.008 24514153

[B8] Busch N. A. Herrmann C. S. Müller M. M. Lenz D. Gruber T. (2006). A cross-laboratory study of event-related gamma activity in a standard object recognition paradigm. NeuroImage 33, 1169–1177. 10.1016/j.neuroimage.2006.07.034 17023180

[B9] Buschkuehl M. Hernandez-Garcia L. Jaeggi S. M. Bernard J. A. Jonides J. (2014). Neural effects of short-term training on working memory. Cogn. Affect. Behav. Neurosci. 14, 147–160. 10.3758/s13415-013-0244-9 24496717 PMC4058299

[B10] Callaway E. Halliday R. (1982). The effect of attentional effort on visual evoked potential N1 latency. Psychiatry Res. 7, 299–308. 10.1016/0165-1781(82)90066-x 6962438

[B11] Chen Y.-N. Mitra S. (2009). The spatial-verbal difference in the N-back task: an ERP study. Acta Neurol. Taiwan. 18, 170–179. Available online at: https://europepmc.org/article/med/19960960. 19960960

[B12] Chen Y.-N. Mitra S. Schlaghecken F. (2008). Sub-processes of working memory in the N-back task: an investigation using ERPs. Clin. Neurophysiol. 119, 1546–1559. 10.1016/j.clinph.2008.03.003 18448388

[B13] Cohen M. X. (2017). MATLAB for Brain and Cognitive Scientists. Cambridge, MA: MIT Press.

[B15] Collingridge D. S. (2013). A primer on quantized data analysis and permutation testing. J. Mixed Methods Res. 7, 81–97. 10.1177/1558689812454457

[B16] Croft R. J. Barry R. J. (2000). Removal of ocular artifact from the EEG: a review. Neurophysiol. Clin. 30, 5–19. 10.1016/S0987-7053(00)00055-1 10740792

[B17] Danker J. F. Hwang G. M. Gauthier L. Geller A. Kahana M. J. Sekuler R. (2008). Characterizing the ERP old-new effect in a short-term memory task. Psychophysiology 45, 784–793. 10.1111/j.1469-8986.2008.00672.x 18513360 PMC2828935

[B18] De Ribaupierre A. Lecerf T. (2006). Relationships between working memory and intelligence from a developmental perspective: convergent evidence from a neo-Piagetian and a psychometric approach. Eur. J. Cogn. Psychol. 18, 109–137. 10.1080/09541440500216127

[B19] Delorme A. Makeig S. (2004). EEGLAB: an open source toolbox for analysis of single-trial EEG dynamics including independent component analysis. J. Neurosci. Methods 134, 9–21. 10.1016/j.jneumeth.2003.10.009 15102499

[B20] Derrick B. White P. Toher D. (2019). Parametric and non-parametric tests for the comparison of two samples which both include paired and unpaired observations. J. Mod. Appl. Stat. Methods 18, 2–23. 10.22237/jmasm/1556669520

[B21] Dong L. Liu X. Zhao L. Lai Y. Gong D. Liu T.. (2019). A comparative study of different EEG reference choices for event-related potentials extracted by independent component analysis. Front. Neurosci. 13:1068. 10.3389/fnins.2019.01068 31680810 PMC6798171

[B22] Dong S. Reder L. M. Yao Y. Liu Y. Chen F. (2015). Individual differences in working memory capacity are reflected in different ERP and EEG patterns to task difficulty. Brain Res. 1616, 146–156. 10.1016/j.brainres.2015.05.003 25976774

[B23] Efron B. RJ T. (1993). An Introduction to the Bootstrap. New York, NY: Chapman and Hall.

[B24] Farrell J. M. Johnston M. E. Twynam G. D. (1998). Volunteer motivation, satisfaction, and management at an elite sporting competition. J. Sport Manag. 12, 288–300. 10.1123/jsm.12.4.288

[B25] Esposito F. Aragri A. Piccoli T. Tedeschi G. Goebel R. Di Salle F. (2009). Distributed analysis of simultaneous EEG-fMRI time-series: modeling and interpretation issues. Magn. Reson. Imaging 27, 1120–1130. 10.1016/j.mri.2009.01.007 19261423

[B26] Folstein J. R. Van Petten C. (2008). Influence of cognitive control and mismatch on the N2 component of the ERP: a review. Psychophysiology 45, 152–170. 10.1111/j.1469-8986.2007.00602.x 17850238 PMC2365910

[B27] Fox M. D. Snyder A. Z. Vincent J. L. Raichle M. E. (2007). Intrinsic fluctuations within cortical systems account for intertrial variability in human behavior. Neuron 56, 171–184. 10.1016/j.neuron.2007.08.023 17920023

[B28] Glahn D. C. Ragland J. D. Abramoff A. Barrett J. Laird A. R. Bearden C. E.. (2005). Beyond hypofrontality: a quantitative meta-analysis of functional neuroimaging studies of working memory in schizophrenia. Hum. Brain Mapp. 25, 60–69. 10.1002/hbm.20138 15846819 PMC6871703

[B29] Green C. S. Bavelier D. Kramer A. F. Vinogradov S. Ansorge U. Ball K. K. (2019). Improving methodological standards in behavioral interventions for cognitive enhancement. J. Cogn. Enhanc. 3, 2–29. 10.1007/s41465-018-0115-y

[B311] Groppe D. M. Makeig S. Kutas M. (2009). Identifying reliable independent components *via* split-half comparisons. NeuroImage 45, 1199–1211. 19162199 10.1016/j.neuroimage.2008.12.038 PMC3062525

[B30] Guo B. Yuan Y. (2017). A comparative review of methods for comparing means using partially paired data. Stat. Methods Med. Res. 26, 1323–1340. 10.1177/0962280215577111 25834090

[B31] Heishman S. J. Kleykamp B. A. Singleton E. G. (2010). Meta-analysis of the acute effects of nicotine and smoking on human performance. Psychopharmacology 210, 453–469. 10.1007/s00213-010-1848-1 20414766 PMC3151730

[B32] Herff C. Heger D. Fortmann O. Hennrich J. Putze F. Schultz T. (2014). Mental workload during n-back task—quantified in the prefrontal cortex using fNIRS. Front. Hum. Neurosci. 7:935. 10.3389/fnhum.2013.00935 24474913 PMC3893598

[B33] Hillyard S. A. Vogel E. K. Luck S. J. (1998). Sensory gain control (amplification) as a mechanism of selective attention: electrophysiological and neuroimaging evidence. Philos. Trans. R. Soc. Lond. B Biol. Sci. 353, 1257–1270. 10.1098/rstb.1998.0281 9770220 PMC1692341

[B34] Jaeggi S. M. Buschkuehl M. Jonides J. Perrig W. J. (2008). Improving fluid intelligence with training on working memory. Proc. Natl. Acad. Sci. U S A 105, 6829–6833. 10.1073/pnas.0801268105 18443283 PMC2383929

[B35] Jaeggi S. M. Buschkuehl M. Perrig W. J. Meier B. (2010). The concurrent validity of the N-back task as a working memory measure. Memory 18, 394–412. 10.1080/09658211003702171 20408039

[B36] Jaeggi S. M. Buschkuehl M. Shah P. Jonides J. (2014). The role of individual differences in cognitive training and transfer. Mem. Cogn. 42, 464–480. 10.3758/s13421-013-0364-z 24081919

[B37] Jasper H. H. (1958). The 10-20 electrode system of the international federation. Electroencephalogr. Clin. Neurophysiol. 10, 371–375. 10590970

[B38] Jiang X. Bian G.-B. Tian Z. (2019). Removal of artifacts from EEG signals: a review. Sensors 19:987. 10.3390/s19050987 30813520 PMC6427454

[B39] Kane M. J. Conway A. R. A. Miura T. K. Colflesh G. J. (2007). Working memory, attention control, and the N-back task: a question of construct validity. J. Exp. Psychol. Learn. Mem. Cogn. 33, 615–622. 10.1037/0278-7393.33.3.615 17470009

[B40] Kanske P. Plitschka J. Kotz S. A. (2011). Attentional orienting towards emotion: P2 and N400 ERP effects. Neuropsychologia 49, 3121–3129. 10.1016/j.neuropsychologia.2011.07.022 21816167

[B41] Kleiner M. Brainard D. Pelli D. Ingling A. Murray R. Broussard C. (2007). What’s new in psychtoolbox-3? Perception 36, 1–16. 10.1068/v070821

[B42] Krause C. M. Sillanmäki L. Koivisto M. Saarela C. Häggqvist A. Laine M.. (2000). The effects of memory load on event-related EEG desynchronization and synchronization. Clin. Neurophysiol. 111, 2071–2078. 10.1016/s1388-2457(00)00429-6 11068244

[B43] Kunimi M. (2016). Effects of age, gender, and stimulus presentation period on visual short-term memory. J. Women Aging 28, 24–33. 10.1080/08952841.2014.950499 26745456

[B44] Kutas M. Federmeier K. D. (2011). Thirty years and counting: finding meaning in the N400 component of the event-related brain potential (ERP). Annu. Rev. Psychol. 62, 621–647. 10.1146/annurev.psych.093008.131123 20809790 PMC4052444

[B45] Levene H. (1960). “Robust test for equality of variances,” in Contributions to Probability and Statistics. Essays in Honor of Harold Hotelling, ed. Olkin I. (Palo Alto, CA: Stanford University Press), 278–292.

[B46] Liu Y. Zhang D. Ma J. Li D. Yin H. Luo Y. (2013). The attention modulation on timing: an event-related potential study. PLoS One 8:e66190. 10.1371/journal.pone.0066190 23826089 PMC3691215

[B47] Lu Z. L. Williamson S. J. Kaufman L. (1992). Behavioral lifetime of human auditory sensory memory predicted by physiological measures. Science 258, 1668–1670. 10.1126/science.1455246 1455246

[B48] Luck S. J. (2014). An Introduction to the Event-Related Potential Technique. Cambridge, MA: MIT Press.

[B49] Luck S. J. Heinze H. J. Mangun G. R. Hillyard S. A. (1990). Visual event-related potentials index focused attention within bilateral stimulus arrays. II. Functional dissociation of P1 and N1 components. Electroencephalogr. Clin. Neurophysiol. 75, 528–542. 10.1016/0013-4694(90)90139-b 1693897

[B50] Mecklinger A. (2000). Interfacing mind and brain: a neurocognitive model of recognition memory. Psychophysiology 37, 565–582. 10.1111/1469-8986.3750565 11037034

[B51] Mecklinger A. Pfeifer E. (1996). Event-related potentials reveal topographical and temporal distinct neuronal activation patterns for spatial and object working memory. Cogn. Brain Res. 4, 211–224. 10.1016/s0926-6410(96)00034-1 8924049

[B52] Mencarelli L. Neri F. Momi D. Menardi A. Rossi S. Rossi A.. (2019). Stimuli, presentation modality, and load-specific brain activity patterns during N-back task. Hum. Brain Mapp. 40, 3810–3831. 10.1002/hbm.24633 31179585 PMC6865510

[B53] Minear M. Brasher F. Guerrero C. B. Brasher M. Moore A. Sukeena J. (2016). A simultaneous examination of two forms of working memory training: evidence for near transfer only. Mem. Cogn. 44, 1014–1037. 10.3758/s13421-016-0616-9 27129921

[B54] Miyake A. Shah P. (Eds). (1999). Models of Working Memory: Mechanisms of Active Maintenance and Executive Control. Cambridge, MA: Cambridge University Press.

[B55] Mohammed S. Flores L. Deveau J. Hoffing R. C. Phung C. Parlett C. M.. (2017). The benefits and challenges of implementing motivational features to boost cognitive training outcome. J. Cogn. Enhanc. 1, 491–507. 10.1007/s41465-017-0047-y 30221244 PMC6136448

[B56] Nystrom L. E. Braver T. S. Sabb F. W. Delgado M. R. Noll D. C. Cohen J. D. (2000). Working memory for letters, shapes, and locations: fMRI evidence against stimulus-based regional organization in human prefrontal cortex. NeuroImage 11, 424–446. 10.1006/nimg.2000.0572 10806029

[B57] Oberauer K. (2005). Binding and inhibition in working memory: individual and age differences in short-term recognition. J. Exp. Psychol. Gen. 134, 368–387. 10.1037/0096-3445.134.3.368 16131269

[B58] Olichney J. M. Van Petten C. Paller K. A. Salmon D. P. Iragui V. J. Kutas M. (2000). Word repetition in amnesia: electrophysiological measures of impaired and spared memory. Brain 123, 1948–1963. 10.1093/brain/123.9.1948 10960058

[B59] Owen A. M. McMillan K. M. Laird A. R. Bullmore E. (2005). N-back working memory paradigm: a meta-analysis of normative functional neuroimaging studies. Hum. Brain Mapp. 25, 46–59. 10.1002/hbm.20131 15846822 PMC6871745

[B60] Pahor A. Jaušovec N. (2018). The effects of theta and gamma tACS on working memory and electrophysiology. Front. Hum. Neurosci. 11:651. 10.3389/fnhum.2017.00651 29375347 PMC5767723

[B61] Patel S. H. Azzam P. N. (2005). Characterization of N200 and P300: selected studies of the event-related potential. Int. J. Med. Sci. 2, 147–154. 10.7150/ijms.2.147 16239953 PMC1252727

[B62] Pergher V. Shalchy M. A. Pahor A. Van Hulle M. M. Jaeggi S. M. Seitz A. R. (2019a). Divergent research methods limit understanding of working memory training. J. Cogn. Enhanc. 4, 100–120. 10.1007/s41465-019-00134-7 PMC8336689 34355115

[B63] Pergher V. Wittevrongel B. Tournoy J. Schoenmakers B. Van Hulle M. M. (2019b). Mental workload of young and older adults gauged with ERPs and spectral power during N-Back task performance. Biol. Psychol. 146:107726. 10.1016/j.biopsycho.2019.107726 31276755

[B64] Pergher V. Wittevrongel B. Tournoy J. Schoenmakers B. Van Hulle M. M. (2018). N-back training and transfer effects revealed by behavioral responses and EEG. Brain Behav. 8:e01136. 10.1002/brb3.1136 30350357 PMC6236237

[B65] Pesonen M. Hämäläinen H. Krause C. M. (2007). Brain oscillatory 4–30 Hz responses during a visual N-back memory task with varying memory load. Brain Res. 1138, 171–177. 10.1016/j.brainres.2006.12.076 17270151

[B66] Picton T. W. (1992). The P300 wave of the human event-related potential. J. Clin. Neurophysiol. 9, 456–479. 10.1097/00004691-199210000-00002 1464675

[B67] Pliatsikas C. Veríssimo J. Babcock L. Pullman M. Y. Glei D. A. Weinstein M.. (2019). Working memory in older adults declines with age, but is modulated by sex and education. Q. J. Exp. Psychol. 72, 1308–1327. 10.1177/1747021818791994 30012055

[B68] Polich J. (2007). Updating P300: an integrative theory of P3a and P3b. Clin. Neurophysiol. 118, 2128–2148. 10.1016/j.clinph.2007.04.019 17573239 PMC2715154

[B69] Polich J. Kok A. (1995). Cognitive and biological determinants of P300: an integrative review. Biol. Psychol. 41, 103–146. 10.1016/0301-0511(95)05130-9 8534788

[B70] Redick T. S. Lindsey D. R. (2013). Complex span and n-back measures of working memory: a meta-analysis. Psychon. Bull. Rev. 20, 1102–1113. 10.3758/s13423-013-0453-9 23733330

[B71] Robbins K. A. Touryan J. Mullen T. Kothe C. Bigdely-Shamlo N. (2020). How sensitive are EEG results to preprocessing methods: a benchmarking study. IEEE Trans. Neural Syst. Rehabil. Eng. 28, 1081–1090. 10.1109/TNSRE.2020.2980223 32217478

[B72] Ross P. Segalowitz S. J. (2000). An EEG coherence test of the frontal dorsal versus ventral hypothesis in N-back working memory. Brain Cogn. 43, 375–379. Available online at: https://pubmed.ncbi.nlm.nih.gov/10857729/. 10857729

[B73] Rossion B. Joyce C. A. Cottrell G. W. Tarr M. J. (2003). Early lateralization and orientation tuning for face, word, and object processing in the visual cortex. NeuroImage 20, 1609–1624. 10.1016/j.neuroimage.2003.07.010 14642472

[B74] Ruchkin D. S. Johnson R. Jr. Grafman J. Canoune H. Ritter W. (1992). Distinctions and similarities among working memory processes: an event-related potential study. Cogn. Brain Res. 1, 53–66. 10.1016/0926-6410(92)90005-c 15497435

[B75] Scharinger C. Soutschek A. Schubert T. Gerjets P. (2015). When flanker meets the N-back: what EEG and pupil dilation data reveal about the interplay between the two central-executive working memory functions inhibition and updating. Psychophysiology 52, 1293–1304. 10.1111/psyp.12500 26238380

[B76] Scharinger C. Soutschek A. Schubert T. Gerjets P. (2017). Comparison of the working memory load in n-back and working memory span tasks by means of eeg frequency band power and p300 amplitude. Front. Hum. Neurosci. 11:6. 10.3389/fnhum.2017.00006 28179880 PMC5263141

[B77] Shapiro S. S. Francia R. S. (1972). An approximate analysis of variance test for normality. J. Am. Stat. Assoc. 67, 215–216. 10.1080/01621459.1972.10481232

[B78] Smith N. J. Kutas M. (2015). Regression-based estimation of ERP waveforms: II. Nonlinear effects, overlap correction, and practical considerations. Psychophysiology 52, 169–181. 10.1111/psyp.12320 25195691 PMC5306445

[B79] Van Petten C. Kutas M. (1990). Interactions between sentence context and word frequencyinevent-related brainpotentials. Mem. Cogn. 18, 380–393. 10.3758/bf03197127 2381317

[B800] van Vliet M. Manyakov N. V. Storms G. Fias W. Wiersema J. R. Van Hulle M. M. (2014). Response-related potentials during semantic priming: the effect of a speeded button response task on ERPs. PLoS One 9:e87650. 10.1371/journal.pone.0087650 24516556 PMC3916390

[B80] van Vliet M. Chumerin N. De Deyne S. Wiersema J. R. Fias W. Storms G.. (2015). Single-trial ERP component analysis using a spatiotemporal lcmv beamformer. IEEE Trans. Biomed. Eng. 63, 55–66. 10.1109/TBME.2015.2468588 26285053

[B81] Vogel E. K. Luck S. J. (2000). The visual N1 component as an index of a discrimination process. Psychophysiology 37, 190–203. 10.1111/1469-8986.3720190 10731769

[B82] Watter S. Geffen G. M. Geffen L. B. (2001). The N-back as a dual-task: P300 morphology under divided attention. Psychophysiology 38, 998–1003. 10.1111/1469-8986.3860998 12240676

[B83] Wittevrongel B. Van Hulle M. M. (2016). Faster p300 classifier training using spatiotemporal beamforming. Int. J. Neural Syst. 26:1650014. 10.1142/S0129065716500143 26971787

[B84] Yao D. Qin Y. Hu S. Dong L. Vega M. L. B. Sosa P. A. V. (2019). Which reference should we use for EEG and ERP practice? Brain Topogr. 32, 530–549. 10.1007/s10548-019-00707-x 31037477 PMC6592976

[B85] Zimmer H. D. Cohen R. L. Foley M. A. Guynn M. J. Engelkamp J. Kormi-Nouri R. (2001). Memory for Action: A Distinct form of Episodic Memory? New York, NY: Oxford University Press on Demand.

