# Supplementary material for: N-Back Related ERPs Depend on Stimulus Type, Task Structure, Pre-processing, and Lab Factors
Source: Front Hum Neurosci. 2020 Oct 28;14:549966. doi: 10.3389/fnhum.2020.549966 (PMC7668114; doi:10.3389/fnhum.2020.549966)
Supplement: Supplementary file 1 [file Data_Sheet_1.docx]

Supplementary Material

N-Back related ERPs depend on stimulus type, task structure, pre-processing and lab factors

Mahsa Alizadeh Shalchy^1*#^, Valentina Pergher ^2,3*^, Anja Pahor ^1*^, Marc M. Van Hulle ^2†^, Aaron R. Seitz ^1†#^

*Table S1:* *Mean and SD of accuracy (%) in dataset I (UCR dataset)*

|  | **Task 1** | **Task 2** | **Task 3** |
| --- | --- | --- | --- |
| **words** | *M* = 62.76, *SD* = 14.23 | *M* = 69.53, *SD* = 12.14 | *M* = 78.78, *SD* = 8.20 |
| **pictures** | *M* = 70.52, *SD* = 11.78 | *M* = 73.18, *SD* = 17.11 | *M* = 80.86, *SD* = 14.95 |
| **colors** | *M* = 65.88, *SD* = 13.65 | *M* = 69.01, *SD* = 13.67 | *M* = 80.21, *SD* = 12.55 |

*Table S2: p-values for Stimulus-wise comparison for accuracy (%) in dataset I (UCR dataset)*

|  | **Task 1** | **Task 2** | **Task 3** |
| --- | --- | --- | --- |
| **words vs pictures** | .075 | .232 | .589 |
| **words vs colors** | .493 | .893 | .718 |
| **pictures vs colors** | .199 | .210 | .851 |

*Table S3: p-values for Task-wise comparison for accuracy (%) in dataset I (UCR dataset)*

|  | **Words** | **Pictures** | **Colors** |
| --- | --- | --- | --- |
| **task 1 vs task 2** | .074 | .452 | .441 |
| **task 1 vs task 3** | < .001 | .002 | < .001 |
| **task 2 vs task 3** | < .001 | .067 | .012 |

*
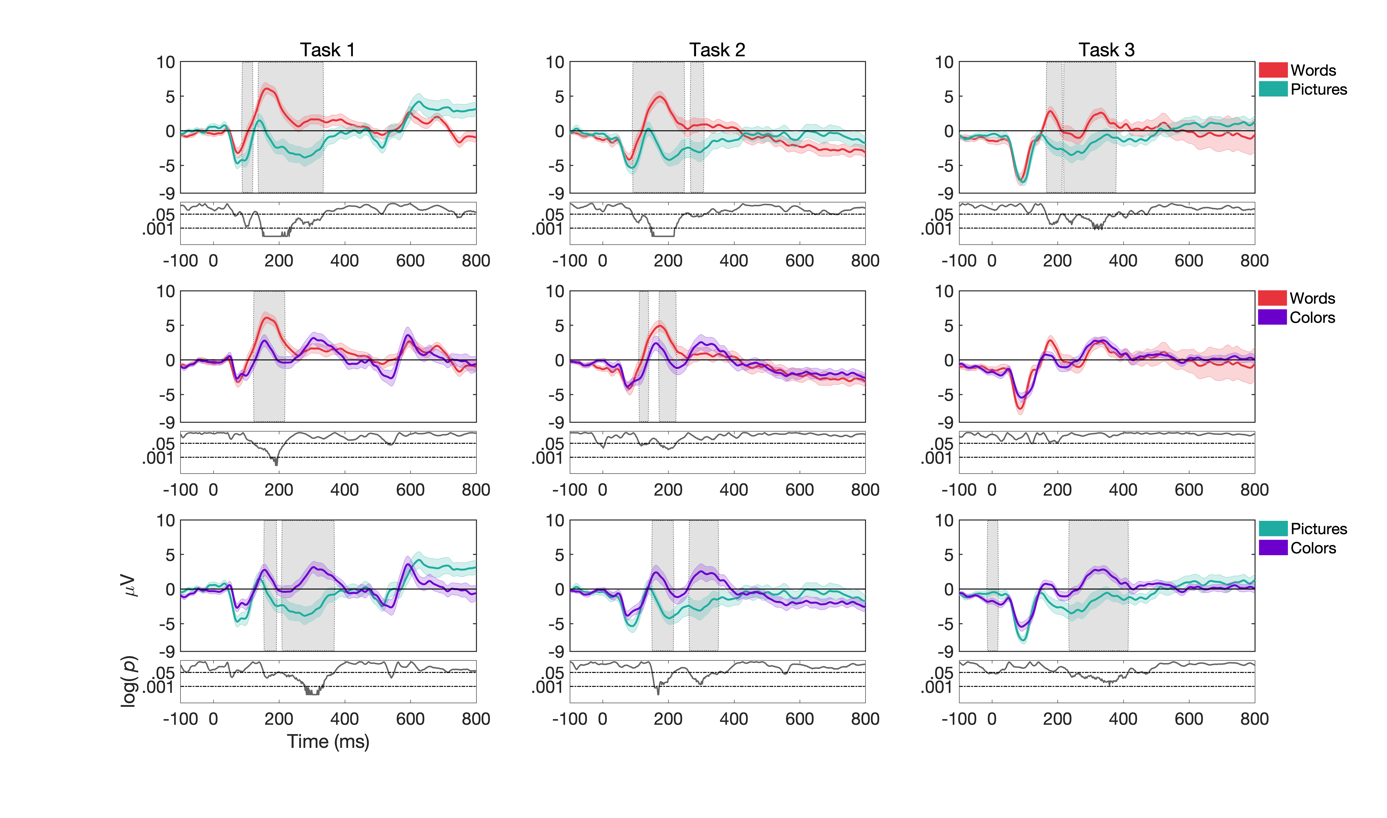
*

*Figure S1: Grand average and SEM of ERP curve at Fz electrode for target trials during variations of stimulus types (words, pictures and colors). The gray shades show p <.05*


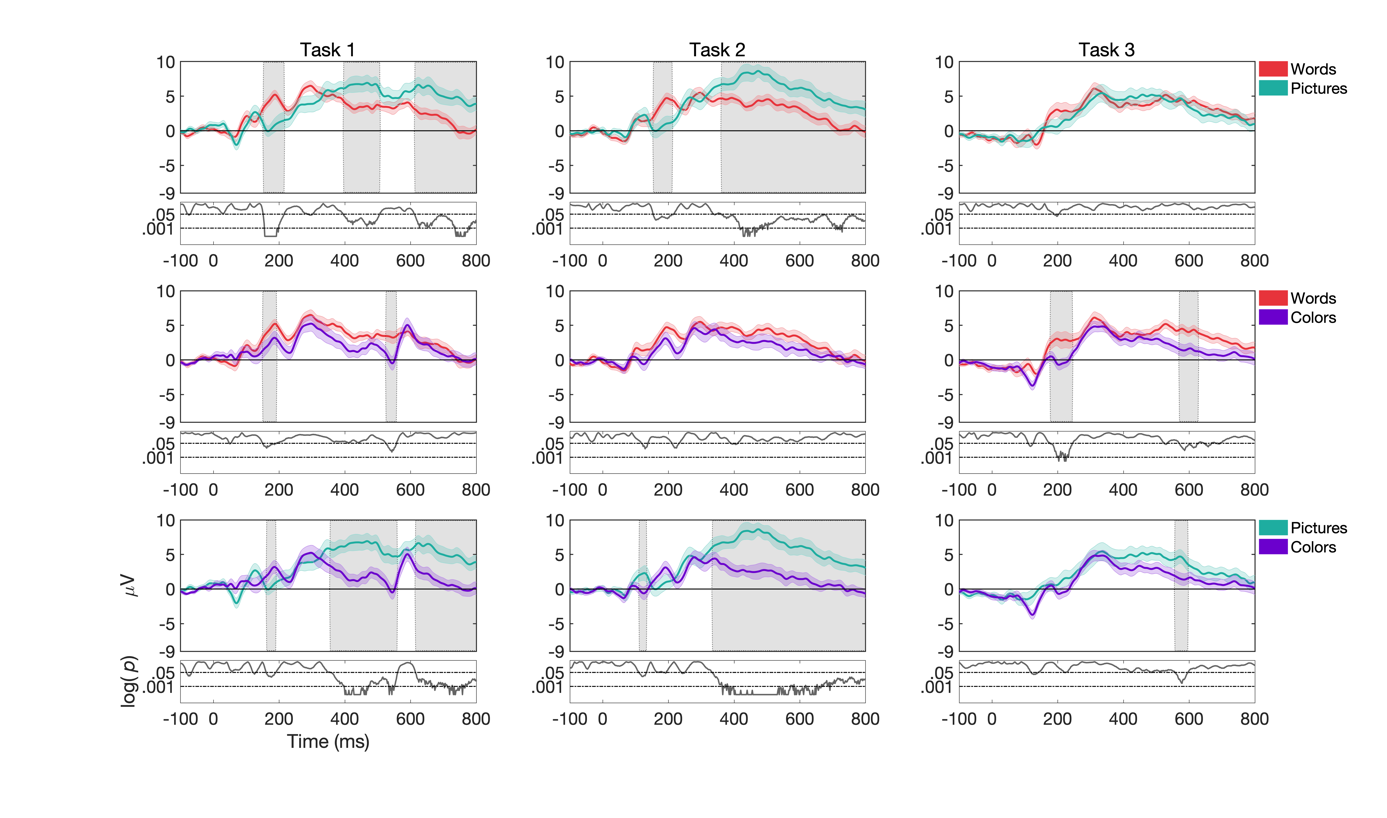


*Figure S2: Grand average and SEM of ERP curve at Pz electrode for target trials during variations of stimulus types (words, pictures and colors). The gray shades show p <.05*


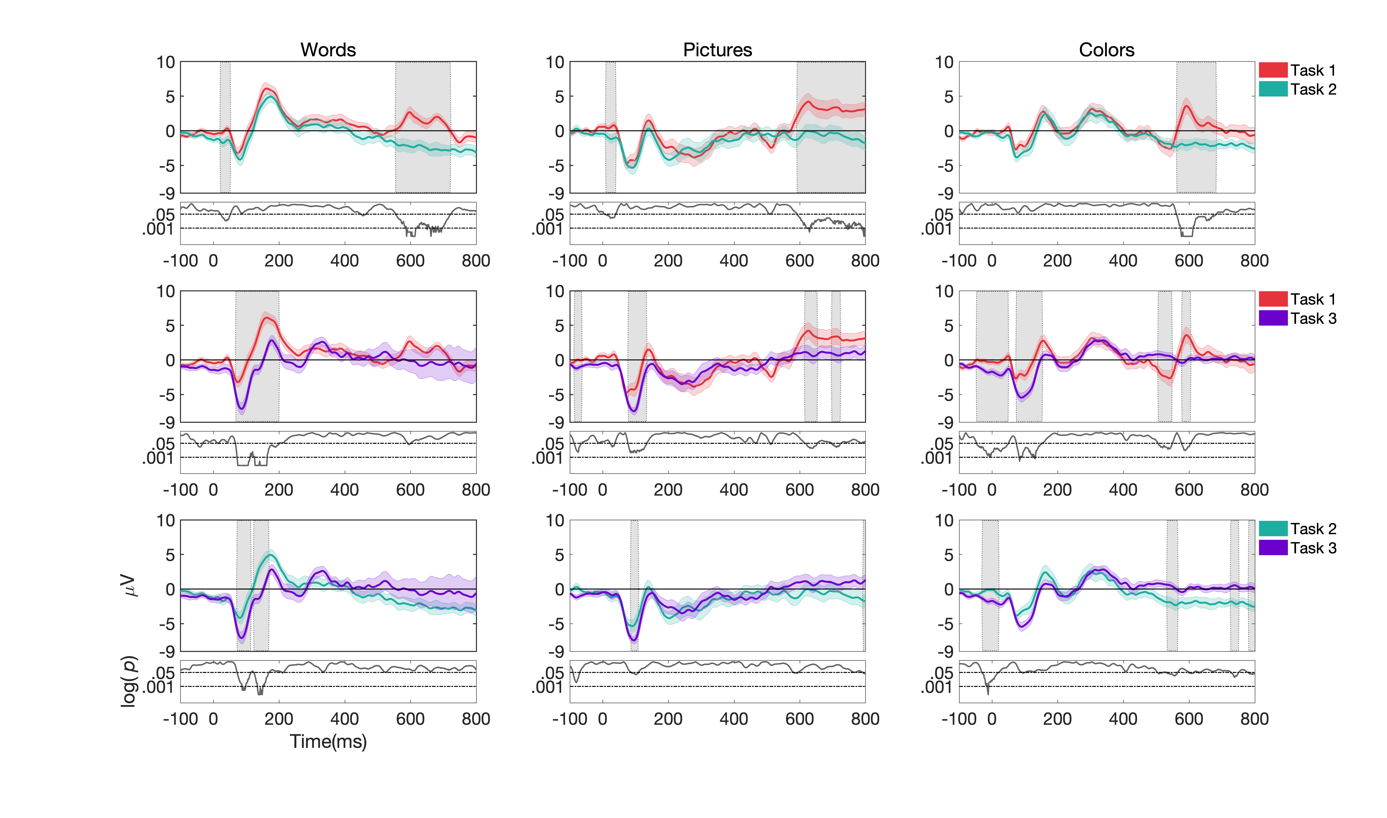


*Figure S3: Grand average and SEM of ERP curve at Fz electrode for target trials during variations of task structure types (task 1, task 2, task 3). The gray shades show p <0.5*


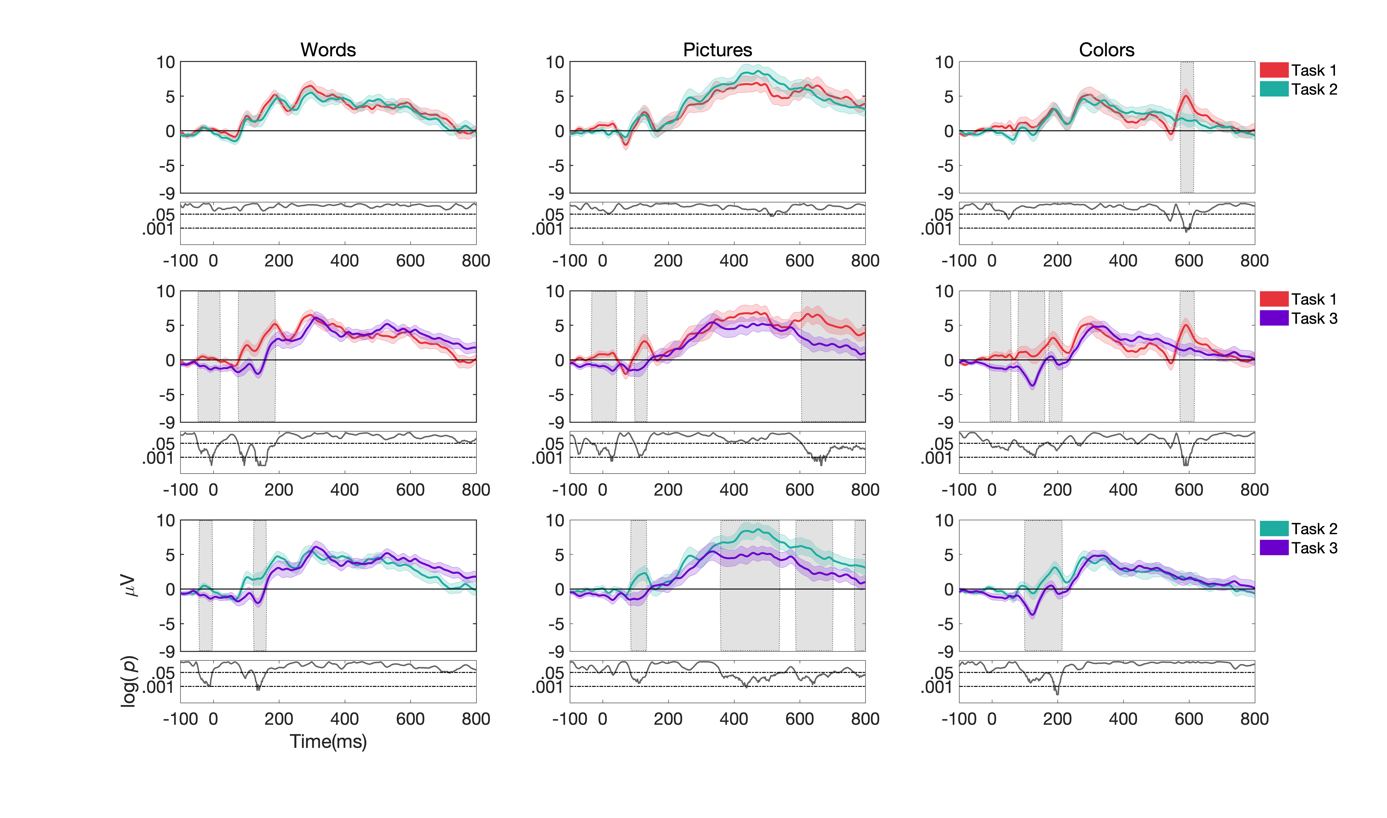


*Figure S4: Grand average and SEM of ERP curve at Pz electrode for target trials during variations of task structure types (task 1, task 2, task 3). The gray shades show p <0.5*

*Table S4: Mixed ANOVA statistics for main and interaction effects of N-back load (N = 2, N = 3)*

|  | P1 | N1 | P2 | N2 | P3 | N4 | PLC |
| --- | --- | --- | --- | --- | --- | --- | --- |
| *task* | <0.001 | <0.001 | <0.005 | 0.488 | 0.929 | 0.984 | 0.017 |
| *stimulus* | 0.163 | <0.001 | <0.001 | <0.001 | 0.362 | 0.008 | <0.001 |
| *load* | 0.181 | 0.031 | <0.001 | <0.001 | <0.001 | <0.001 | <0.001 |
| *task x stimulus* | 0.685 | 0.335 | 0.519 | 0.933 | 0.843 | 0.561 | 0.373 |
| *load x task* | 0.248 | 0.760 | 0.296 | 0.090 | 0.051 | 0.031 | 0.002 |
| *load x stimulus* | 0.762 | 0.867 | 0.576 | 0.468 | 0.971 | 0.661 | 0.883 |
| *load x task x stimulus* | 0.295 | 0.669 | 0.898 | 0.794 | 0.721 | 0.557 | 0.720 |

*
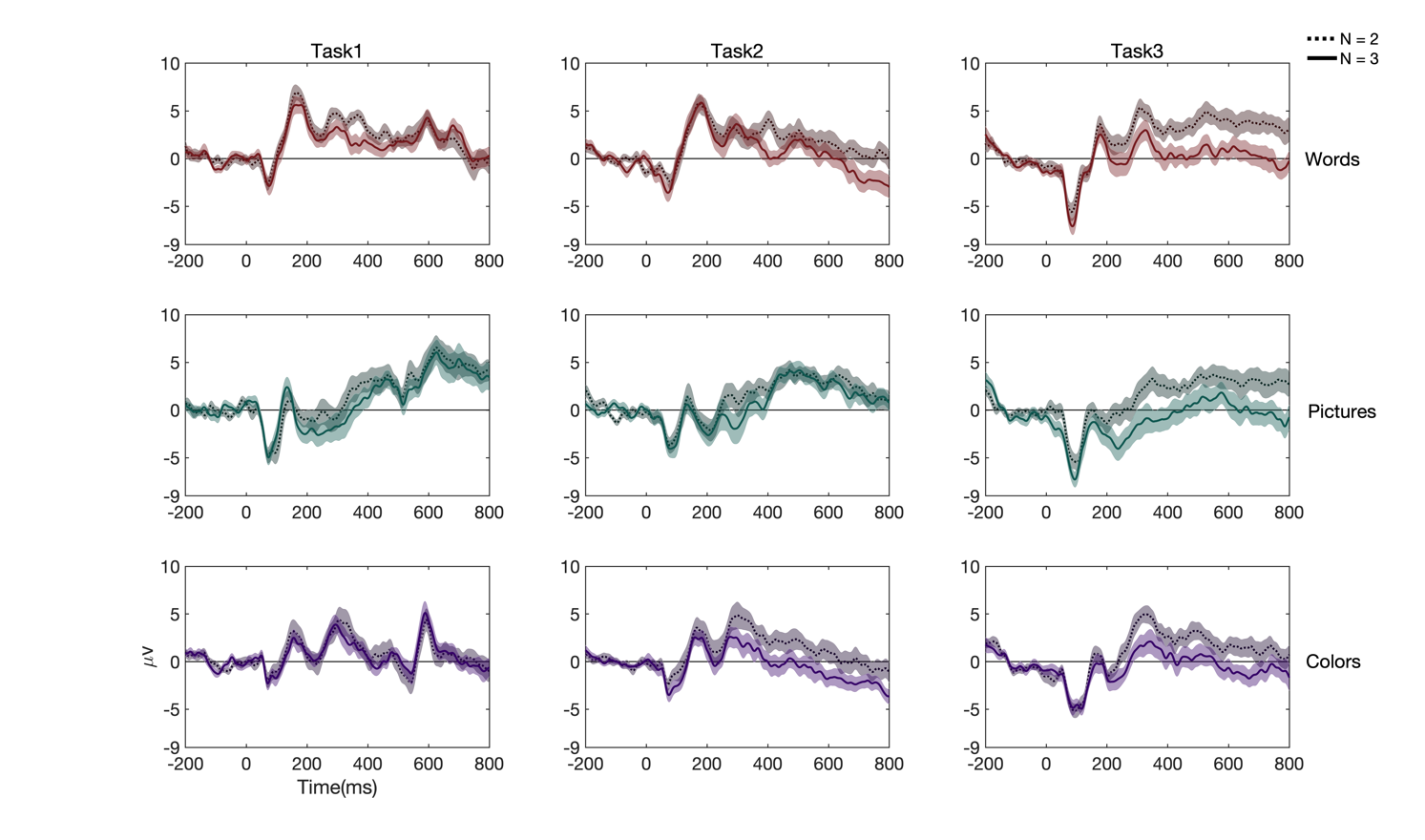
*

*Figure S5. Grand average and SEM of ERP curve at Cz electrode for N-back load (N = 2, N = 3) across task structures and stimulus types*

*Table S5: Mixed ANOVA statistics for main and interaction effects of performance metrics (hits, misses, correct rejection, and false alarm)*

|  | P1 | N1 | P2 | N2 | P3 | N4 | PLC |
| --- | --- | --- | --- | --- | --- | --- | --- |
| *task* | <0.001 | <0.001 | <0.001 | 0.029 | 0.030 | 0.049 | <0.001 |
| *stimulus* | 0.006 | <0.001 | <0.001 | <0.001 | 0.148 | 0.015 | <0.001 |
| *performance* | 0.712 | 0.001 | <0.001 | <0.001 | <0.001 | <0.001 | <0.001 |
| *task x stimulus* | 0.965 | 0.767 | 0.517 | 0.986 | 0.83 | 0.559 | 0.741 |
| *performance x task* | 0.739 | 0.236 | 0.432 | 0.121 | 0.031 | 0.07 | 0.029 |
| *performance x stimulus* | 0.376 | 0.708 | 0.209 | 0.025 | 0.004 | 0.011 | 0.003 |
| *performance x task x stimulus* | 0.946 | 0.368 | 0.780 | 0.600 | 0.53 | 0.49 | 0.652 |


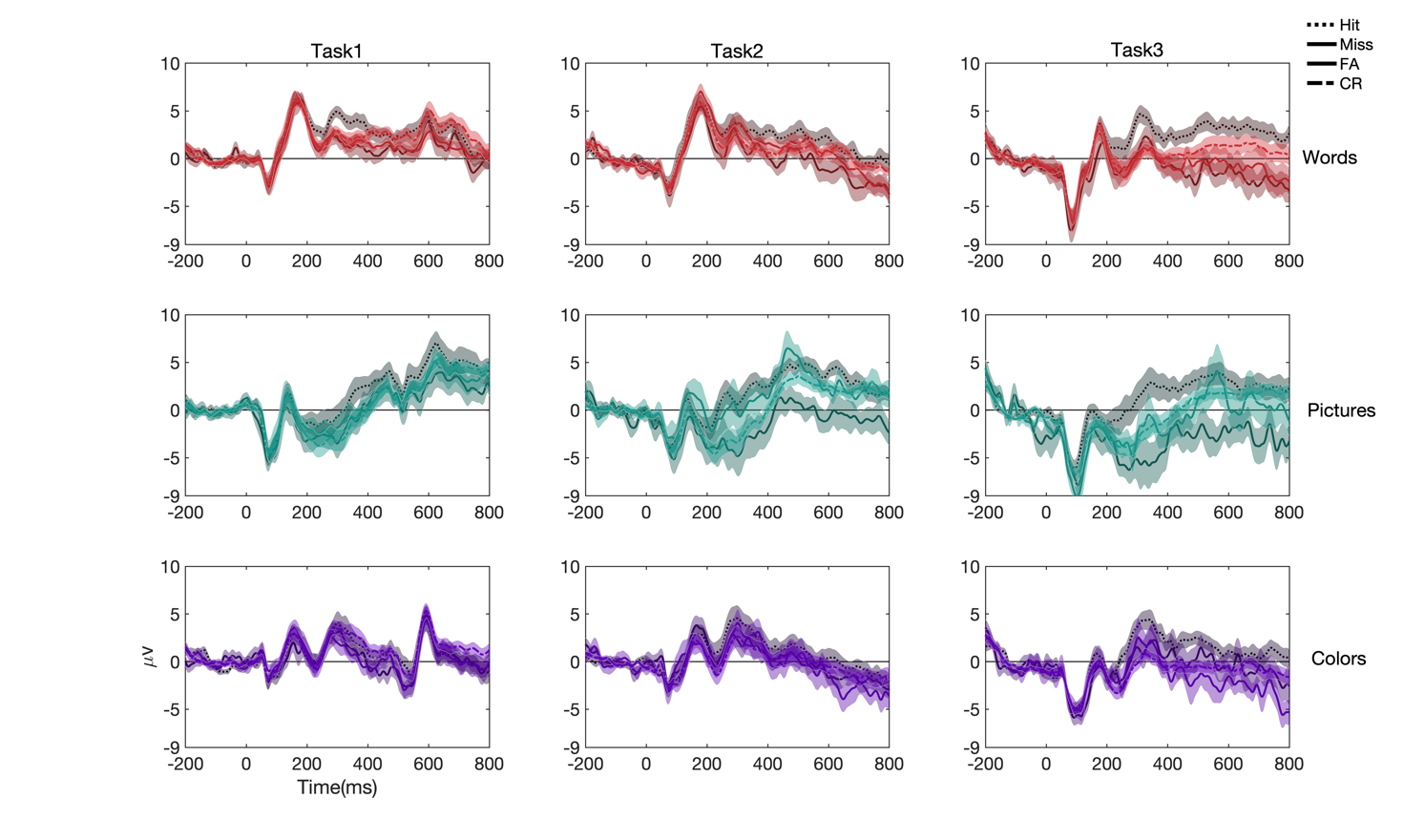


*Figure S6. Grand average and SEM of ERP curve at Cz electrode for performance metrics (hit, miss, correct rejection, and false alarm) across task structures and stimulus types*


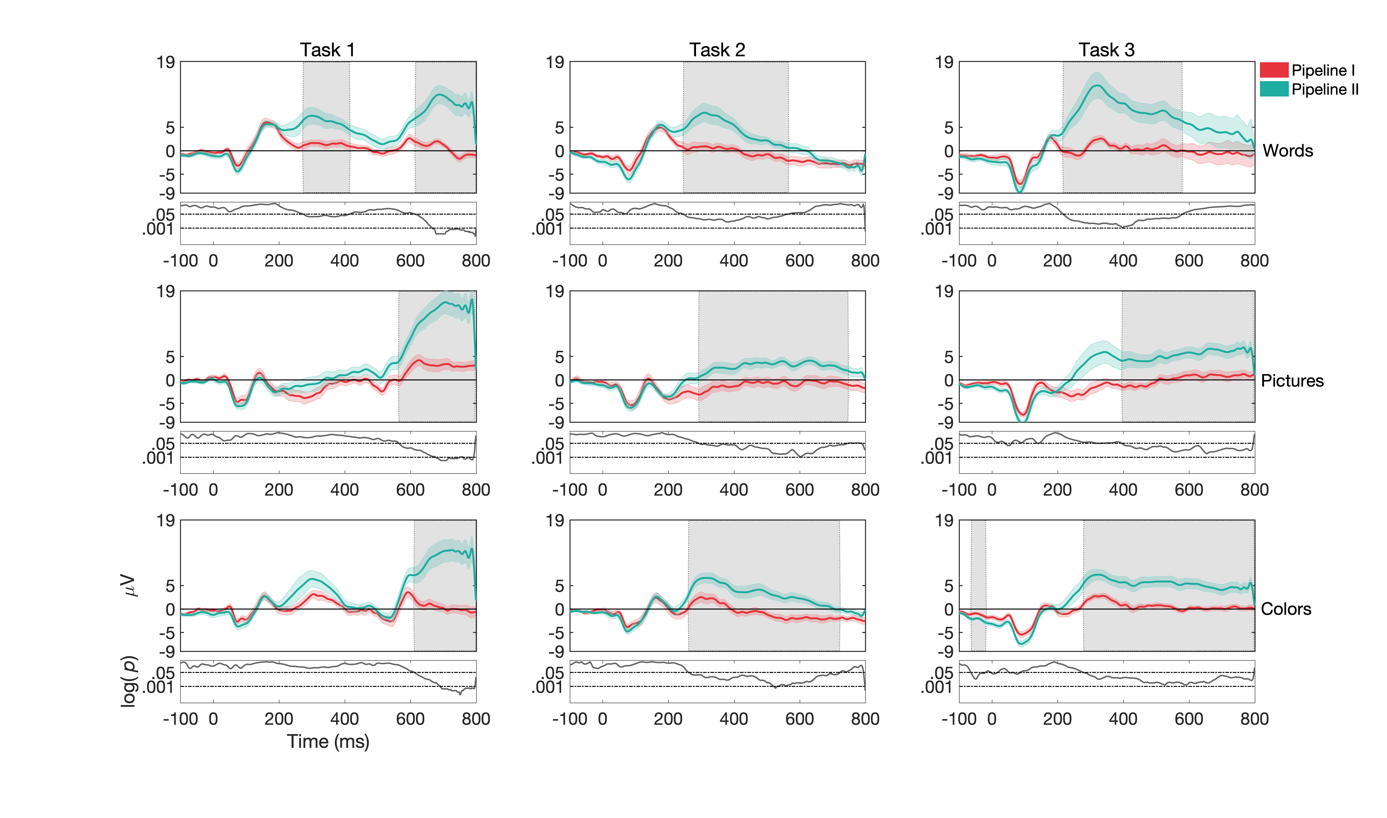


*Figure S7: Grand average and SEM of ERP curve at Fz electrode for target trials for different pipelines (Pipeline I vs. Pipeline II) for dataset I (UCR dataset)*


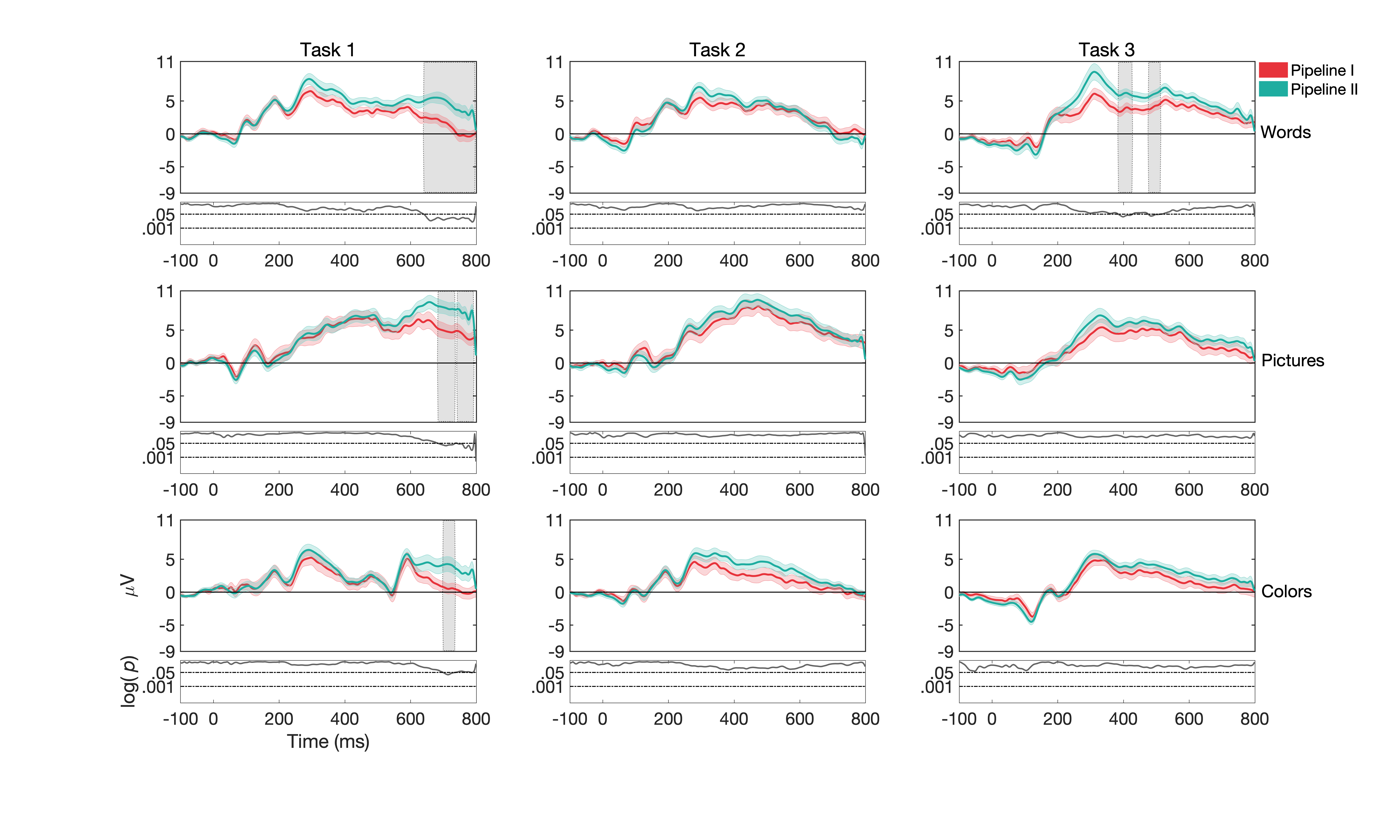


*Figure S8: Grand average and SEM of ERP curve at Pz electrode for target trials for different pipelines (Pipeline I vs. Pipeline II) for dataset I (UCR dataset)*


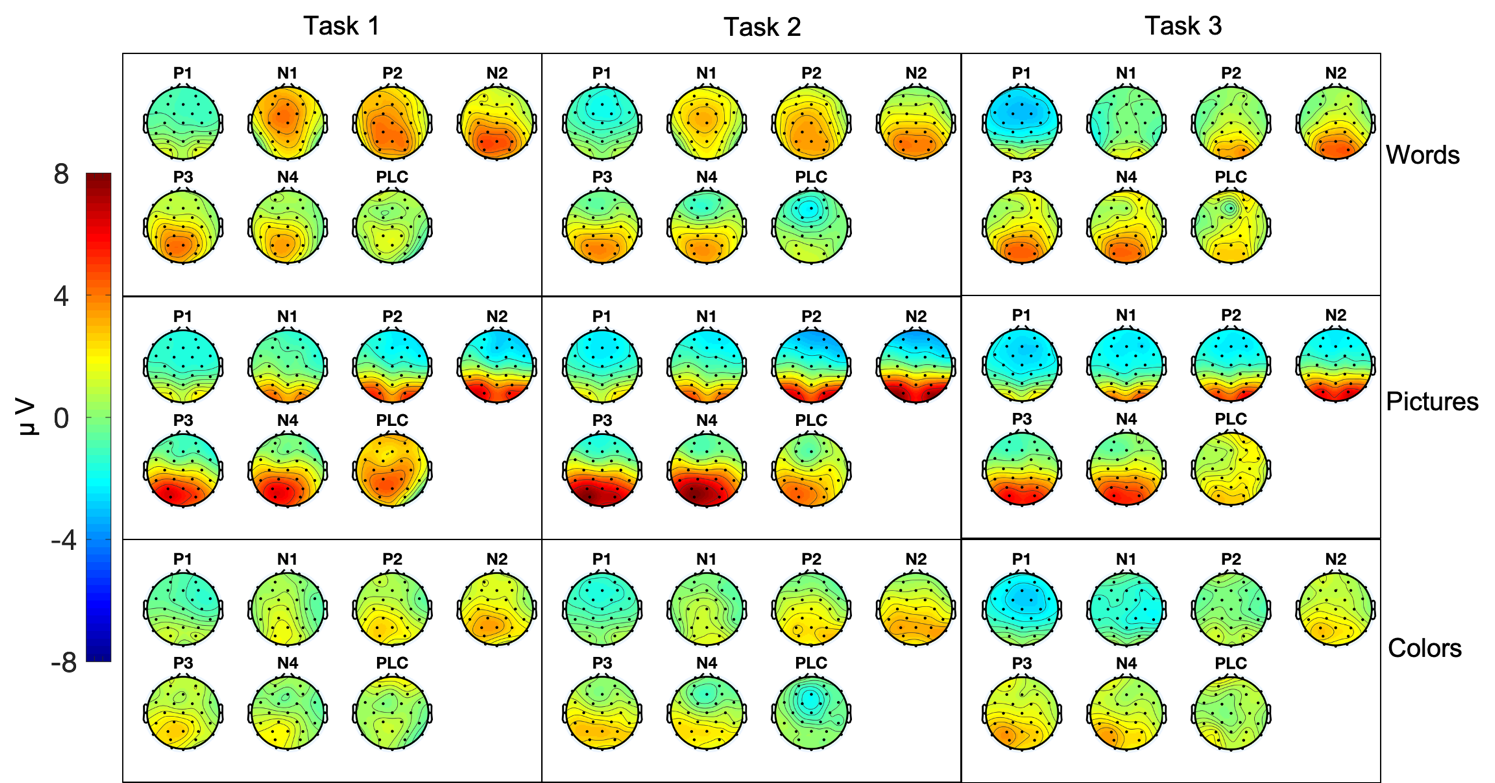


*Figure S9: Topographical maps for dataset I (UCR dataset). The time window for each component is as follows: P1 = [0 100], N1 = [100 200], P2 = [150 275], N2 = [200 350], P3 = [250 600], N4 = [400 600], PLC = [500 1000]*

*Table S6. p-values for different components for effect of condition x electrodes using Kruskal Wallis test*

|  |  | **task 1** | **task 2** | **task 3** |
| --- | --- | --- | --- | --- |
| **words**  **vs**  **pictures** | **P1** | .471 | <.001 | .991 |
|  | **N1** | <.001 | <.001 | <.001 |
|  | **P2** | <.001 | <.001 | <.001 |
|  | **N2** | <.001 | <.001 | <.001 |
|  | **P3** | <.001 | <.001 | <.001 |
|  | **N4** | <.001 | .235 | .055 |
|  | **PLC** | .126 | .998 | .999 |
| **words**  **vs**  **colors** | **P1** | .896 | .999 | .999 |
|  | **N1** | .053 | .698 | .008 |
|  | **P2** | .094 | .991 | .002 |
|  | **N2** | .005 | .995 | .001 |
|  | **P3** | .214 | .999 | .024 |
|  | **N4** | .042 | .972 | .028 |
|  | **PLC** | .822 | .991 | .018 |
| **pictures**  **vs**  **colors** | **P1** | <.001 | .064 | .159 |
|  | **N1** | .007 | <.001 | <.001 |
|  | **P2** | <.001 | <.001 | <.001 |
|  | **N2** | <.001 | <.001 | <.001 |
|  | **P3** | <.001 | <.001 | <.001 |
|  | **N4** | .003 | <.001 | <.001 |
|  | **PLC** | .318 | .440 | .031 |

*Table S7. p-values for different components for effect of condition x electrodes using Kruskal Wallis test*

|  |  | **words** | **pictures** | **colors** |
| --- | --- | --- | --- | --- |
| **task 1**  **vs**  **task 2** | **P1** | .998 | .249 | .979 |
|  | **N1** | .999 | .758 | .914 |
|  | **P2** | .999 | .097 | .598 |
|  | **N2** | .982 | <.001 | .101 |
|  | **P3** | .179 | <.001 | .316 |
|  | **N4** | .002 | <.001 | .601 |
|  | **PLC** | .009 | <.001 | .092 |
| **task 1**  **vs**  **task 3** | **P1** | .001 | .011 | .232 |
|  | **N1** | <.001 | .011 | .323 |
|  | **P2** | .048 | .040 | .854 |
|  | **N2** | .785 | .263 | .999 |
|  | **P3** | .897 | .528 | .999 |
|  | **N4** | .752 | .579 | .993 |
|  | **PLC** | .215 | .029 | .699 |
| **task 2**  **vs**  **task 3** | **P1** | .796 | .973 | .877 |
|  | **N1** | .499 | .999 | .944 |
|  | **P2** | .638 | .999 | .144 |
|  | **N2** | .568 | .999 | .058 |
|  | **P3** | .265 | .999 | .744 |
|  | **N4** | .610 | .987 | .942 |
|  | **PLC** | .339 | .982 | .854 |
